# New insights into the evolution and functional divergence of the SWEET family in *Saccharum* based on comparative genomics

**DOI:** 10.1186/s12870-018-1495-y

**Published:** 2018-11-07

**Authors:** Weichang Hu, Xiuting Hua, Qing Zhang, Jianping Wang, Qiaochu Shen, Xingtan Zhang, Kai Wang, Qingyi Yu, Yann-Rong Lin, Ray Ming, Jisen Zhang

**Affiliations:** 1 Center for Genomics and Biotechnology, Haixia Institute of Science and Technology, Fujian Provincial Key Laboratory of Haixia Applied Plant Systems Biology, College of Life Sciences, Fujian Agriculture and Forestry University, Fuzhou, 350002 China; 2 Key Laboratory of Sugarcane Biology and Genetic Breeding, Ministry of Agriculture, Fujian Agriculture and Forestry University, Fuzhou, 350002 China; 3 Agronomy Department, University of Florida, Gainesville, FL 32610 USA; 4 Texas A&M AgriLife Research, Department of Plant Pathology and Microbiology, Texas A&M University System, Dallas, TX 75252 USA; 5 Department of Agronomy, National Taiwan University, Taipei, 100 Taiwan; 6 Department of Plant Biology, University of Illinois at Urbana-Champaign, Urbana, IL 61801 USA

**Keywords:** Gene expression, Gene evolution, *Saccharum officinarum*, *Saccharum spontaneum*, Sugar transport, SWEET

## Abstract

**Background:**

The SWEET (Sugars Will Eventually be Exported Transporters) gene family is a recently identified group of sugar transporters that play an indispensable role in sugar efflux, phloem loading, plant-pathogen interaction, nectar secretion, and reproductive tissue development. However, little information on *Saccharum SWEET* is available for this crop with a complex genetic background.

**Results:**

In this study, 22 *SWEET* genes were identified from *Saccharum spontaneum* Bacterial Artificial Chromosome libraries sequences. Phylogenetic analyses of *SWEETs* from 11 representative plant species showed that gene expansions of the SWEET family were mainly caused by the recent gene duplication in dicot plants, while these gene expansions were attributed to the ancient whole genome duplication (WGD) in monocot plant species. Gene expression profiles were obtained from RNA-seq analysis. *SWEET1a* and *SWEET2s* had higher expression levels in the transitional zone and maturing zone than in the other analyzed zones. *SWEET1b* was mainly expressed in the leaf tissues and the mature zone of the leaf of both *S. spontaneum* and *S. officinarum*, and displayed a peak in the morning and was undetectable in both sclerenchyma and parenchyma cells from the mature stalks of *S. officinarum*. *SsSWEET4a*\*4b* had higher expression levels than *SWEET4c* and were mainly expressed in the stems of seedlings and mature plants. *SWEET13s* are recently duplicated genes, and the expression of *SWEET13s* dramatically increased from the maturing to mature zones. *SWEET16b*’s expression was not detected in *S. officinarum*, but displayed a rhythmic diurnal expression pattern.

**Conclusions:**

Our study revealed the gene evolutionary history of SWEETs in *Saccharum* and *SWEET1b* was found to be a sucrose starvation-induced gene involved in the sugar transportation in the high photosynthetic zones. *SWEET13c* was identified as the key player in the efflux of sugar transportation in mature photosynthetic tissues. *SWEET4a*\*4b* were found to be mainly involved in sugar transportation in the stalk. *SWEET1a\2a\4a\4b\13a\16b* were suggested to be the genes contributing to the differences in sugar contents between *S. spontaneum* and *S. officinarum*. Our results are valuable for further functional analysis of *SWEET* genes and utilization of the *SWEET* genes for genetic improvement of *Saccharum* for biofuel production.

**Electronic supplementary material:**

The online version of this article (10.1186/s12870-018-1495-y) contains supplementary material, which is available to authorized users.

## Background

In mesophyll cells, carbon fixation allows the production of sugars by photosynthesis. Sugars are not only key energy sources for many biological activities in plants and human, but also function as important signaling molecules [1]. In plants, sugars are transported from prototrophic tissues (leaves, source) to heterotrophic cells or tissues (seeds, sink) via plasmodesmata or the apoplastic pathway. There are three gene families of transporters that play a key role in the intercellular transport of sugars: *MonoSaccharide Transporters* (*MSTs*), *Sucrose Transporters/SucroseCarriers* (*SUTs*) [2–5], and *SWEETs* [6–8]. The first two gene families of sugar transporter, *MSTs* and *SUTs*, have been extensively studied in higher plants in the last two decades [4, 9–16], whereas the *SWEET* gene family was recently identified as sugar effluxers [7] based the role of its members in the transport of hexose or sucrose across cell membranes.

The first member of the MtN3/saliva/SWEET gene family, *MtN3*, was identified as nodulin in the interaction between the legume *Medicago truncatula* and *Rhizobium* during nodule development [17], and another SWEET-type gene, *saliva*, was identified in *Drosophila* as a salivary gland specific gene during embryonic development [18]. Since 2010, *SWEETs* and their prokaryotic homologues have been identified in various organisms, spanning from Archaea to plants and humans, based on their sugar transporter function [6, 8, 19–21]. In rice, the paralogous *OsSWEET11/Os8N3/Xa13* and *OsSWEET14/Xa25* are targeted by the pathogenic bacterium *Xanthomonas oryzae pv. oryzae* (*Xoo*) and hijacked for nutritional gain to support their own growth, which results in bacterial blight [22]. A more recent study showed that both rice *OsSWEET4* and its maize ortholog *ZmSWEET4c* encode for hexose transporters, and the mutants of these two genes are defective in seed filling, indicating that a lack of hexose transport in the basal endosperm transfer layer (BETL) fails to sustain development of the large starch-storing endosperm of cereal grains [19]. In Arabidopsis, AtSWEET11 and AtSWEET12 localize to the plasma membrane of the phloem parenchyma cells. Analysis of *atsweet11/12* double mutant plants showed an impaired ability to export sucrose from leaves [8]. AtSWEET16 and AtSWEET17 have been identified as vacuolar hexose transporters controlling leaf fructose content [20, 21]. AtSWEET9 was shown to be a nectary-specific sugar transporter and responsible for nectar production [6]. Eom [23] and Chen [24] recently reviewed the function of the SWEET family proteins in diverse biological and physiological processes including phloem loading, leaf and senescence fructose conservation, pollen nutrition and seed filling, pathogen susceptibility, nectar secretion.

Sugarcane contributes to approximately 80% of the sugar and 40% of ethanol world production [25]. Remarkably, sugarcane can accumulate vast amounts of sucrose up to 700 mM or more than 50% of the dry weight (DW) in its stems [26]. Modern sugarcane cultivars are hybrids derived from the cross between *S. officinarum* and *S. spontaneum*, resulting in extreme allopolyploidy levels that can range from octoploidy (x = 8) to dodecaploidy (x = 12). The genetic background for sugar accumulation of modern sugarcane cultivars has been suggested to be conferred by *S. officinarum*, while stress tolerance was attributed to *S. spontaneum*. Sucrose photosynthesized in the sugarcane leaf (source) is transported to phloem parenchyma cells for loading [27]. Allocation of sucrose is facilitated by long and short-distance transport systems [28, 29]. Ultimately, it is unloaded from the phloem and imported into parenchyma cells in the stem (sink) storage tissue for further metabolism. During the whole process, some of the sucrose is exported into the cell wall, where is hydrolyzed by invertases into fructose and glucose to sustain growth at specific sites [30, 31], demonstrating that the apoplast solution of sugarcane stalk tissues contains high concentrations of sucrose. Recently, the gene families related to sugar accumulation in sugarcane have been extensively studied [32–36].

In spite of the great significance of *SWEETs* in sugarcane, the *SWEET* gene family had not been identified in sugarcane until now because sugarcane genome information was unavailable, and *SWEETs* are a recently identified class of sugar transporters. In this study, given the multiple roles of *SWEETs* particularly in sugar transport and pathogen susceptibility, based on our high coverage BAC libraries, we investigated the *SWEET* genes in *Saccharum*. To understand the molecular and evolutionary characterization and gene functions of the *SWEET* family in sugarcane, we investigated the phylogenetic relationships among different species, analyzed exon/intron organization, and gene expression. The results presented here provide a reference for further studies on the biological and physiological functions of *SWEET* genes, and shed light on the mechanisms of sugar accumulation in sugarcane.

## Results

### Identification of *SWEET* genes in sugarcane and other species

**Fig. 1:**
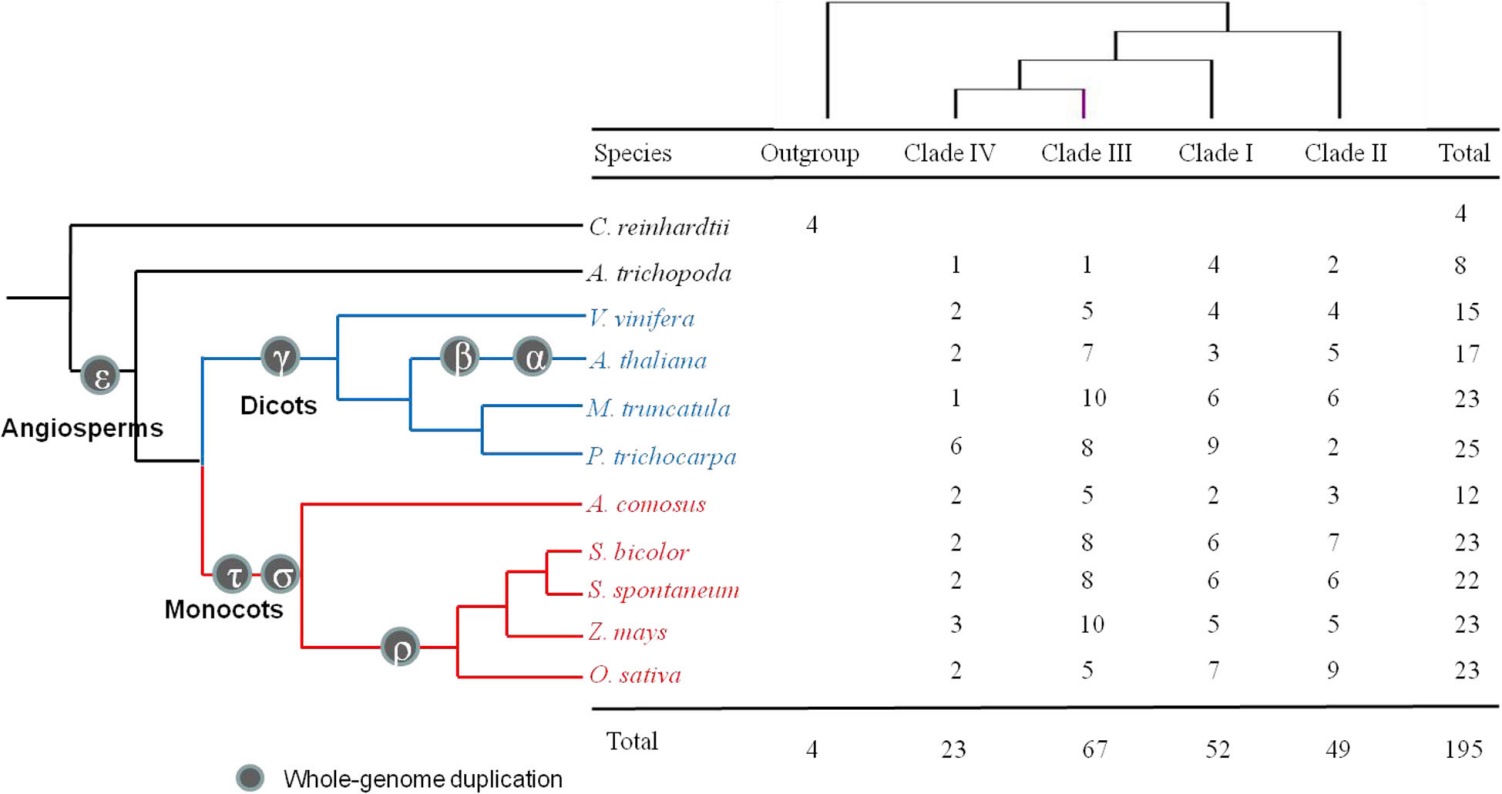
Phylogenetic relationships of *SWEET* families based on the current data for angiosperms were used in this study [94]. The number of *SWEET* genes found in the genome of each species is indicated

Based on the reference sequences of *SWEETs* from previous studies in *Oryza sativa* [37] and *Arabidopsis thaliana* [38]*,* the *SWEET* genes were searched from 10 representative plant genomes downloaded from phytozome 10 [39]. Moreover, the hidden Markov model-based HMMER program [40] was run for the 10 representative plant genomes, confirming 173 *SWEET* genes with high homology (Fig. 1), which included 4 in *Chlamydomonas reinhardtii*, 8 in *Amborella trichopoda*, 15 in *Vitis vinifera*, 17 in *Arabidopsis thaliana*, 23 in *Medicago truncatula*, 25 in *Populus trichocarpa*,12 in *Ananus comosus*, 23 in *Sorghum bicolor*, 23 in *Zea mays*, and 23 in *Oryza sativa*.

Using the protein sequences of sorghum *SWEET* genes as a reference, we identified 22 *S. spontaneum SWEET* haplotypes, excluding gene alleles from the high coverage BAC sequence database. The recently published tetraploidy *S. spontaneum* genome [41] combined with the sorghum genome were further used as reference, each of these genes were observed to have an average of 3 allelic haplotypes in the *S. spontaneum* genome. Of the 22 *SWEET* gene members in *S. spontaneum*, two genes(*SsSWEET12*, *SsSWEET16a*), were located on chromosome 1, four genes (*SsSWEET11b*, *SsSWEET13a*, *SsSWEET13b*, *SsSWEET13c*) were located on chromosome 2, seven genes (*SsSWEET1a*, *SsSWEET2a*, *SsSWEET2b*, *SsSWEET3b*, *SsSWEET4d*, *SsSWEET6*, *SsSWEET16b*) were located on chromosome 3, four genes (*SsSWEET4a*, *SsSWEET4b*, *SsSWEET4c*, *SsSWEET15*) were located on chromosome 4, two genes(*SsSWEET11a*, *SsSWEET14*), were located on chromosome 6, and three genes (*SsSWEET1b*, *SsSWEET3a*, *SsSWEET5*) were located on chromosome 7. Noteworthy, the three *SWEET13s* and three of *SsSWEET4s* (*SsSWEET4a*, *SsSWEET4b*, *SsSWEET4c*) were originated from tandem duplications, respectively (Additional file 1). For consistency, *S. spontaneum SWEET* genes were named based on the previously reported *O. sativa SWEET* nomenclature [22, 42] and phylogenetic relationships. If two or more *S. spontaneum* genes were equally close to a single rice gene, then the same name was used followed by the letters “a”, “b”, “c” “d” and “e”. More details about other features, including protein and expressed sequence tags (EST) sequence information, are presented in Table 1. The 22 *S. spontaneum SWEET* genes nucleotide sequences were submitted to GenBank with accession numbers MG204840-MG204861.

**Table 1:** Comparison of the information of the *SWEETs* between *Saccharum spontaneum* and *Sorghum bicolor*

*Sorghum bicolor*						*Saccharum spontaneum*							
Gene	AA	Mw (kDa)	pI	TM	MtN3/saliva domain position	Gene	AA	Mw (kDa)	pI	TM	MtN3/saliva domain position	ESTs	Similarities
*Sb03g041740*	269	29.07	9.36	6	7–96,130–215	*SsSWEET1a*	269	29.23	9.34	6	7–92,130–216	21	93%
*Sb09g020860*	256	27.27	9.12	7	8–95,134–215	*SsSWEET1b*	277	28.69	9.05	8	31–113,152–232	7	94%
*Sb03g024250*	243	26.82	8.86	7	26–108,145–226	*SsSWEET2a*	243	26.95	8.86	7	26–108,145–226	10	98%
*Sb03g032190*	231	25.15	9.18	7	13–99,136–219	*SsSWEET2b*	227	23.09	9.13	6	13–99,156–204	5	88%
*Sb09g006950*	246	27.34	8.75	7	7–98,132–218	*SsSWEET3a*	412	45.31	8.87	7	8–97,143–226	3	75%
*Sb03g001520*	259	28.39	8.94	7	10–98,134–217	*SsSWEET3b*	292	32.16	8.62	6	10–98,162–244	–	83%
*Sb04g015420*	250	27.20	9.36	7	11–94,133–217	*SsSWEET4a*	253	26.49	9.46	7	11–94,133–217	23	98%
*Sb04g012910*	250	27.43	9.54	7	11–94,133–217	*SsSWEET4b*	254	26.78	9.44	7	10–94,133–217	36	97%
*Sb04g012920*	252	27.62	9.38	7	11–92,133–217	*SsSWEET4c*	251	27.61	9.26	7	11–92,133–217	7	93%
*Sb03g003470*	213	23.69	4.53	7	5–87,120–203	*SsSWEET4d*	362	36.87	6.24	6	162–240,279–361	–	33%
*Sb03g003480*	242	26.18	6.87	6	12–95,135–194	*N/A*	*N/A*	*N/A*	*N/A*	*N/A*	*N/A*	*N/A*	*N/A*
*Sb09g030270*	239	25.93	6.81	7	10–97,133–215	*SsSWEET5*	239	24.68	7.62	7	10–97,133–215	–	93%
*Sb03g027260*	244	27.20	9.30	7	10–97,133–216	*SsSWEET6*	291	30.40	9.53	8	10–97,202–263	3	80%
*Sb07g026040*	310	33.52	9.45	7	17–100,136–218	*SsSWEET11a*	308	31.66	9.36	7	17–100,136–219	7	98%
*Sb02g029430*	273	29.75	9.03	7	15–99,136–218	*SsSWEET11b*	286	29.30	9.08	7	15–99,136–220	1	90%
*Sb01g035490*	314	34.39	6.84	7	11–95,133–214	*SsSWEET12*	308	33.72	5.41	7	11–94,132–213	–	93%
*Sb08g014040*	303	33.01	9.50	7	13–99,134–218	*SsSWEET13a*	304	31.57	9.57	7	13–99,134–218	13	95%
*Sb08g013840*	303	33.24	9.74	7	13–99,134–218	*SsSWEET13b*	302	33.14	9.50	7	13–99,134–218	13	93%
*Sb08g013620*	305	33.25	9.48	7	13–98,134–218	*SsSWEET13c*	303	33.11	9.55	7	13–99,134–218	14	97%
*Sb05g018110*	292	31.82	9.21	7	13–101,136–219	*SsSWEET14*	272	28.39	9.12	6	11–79,114–197	8	86%
*Sb04g021000*	338	35.69	7.61	7	14–99,135–219	*SsSWEET15*	327	33.21	5.96	7	14–99,135–219	6	87%
*Sb01g035840*	330	35.19	9.30	7	7–92,129–212	*SsSWEET16a*	320	32.72	9.50	7	7–92,128–212	4	89%
*Sb03g012930*	242	26.38	6.41	6	6–93,129–215	*SsSWEET16b*	240	25.22	6.27	7	7–92,130–214	1	93%

The 22 identified SsSWEET genes contain full open reading frames (ORFs) with predicted molecular weights ranging between 23.09 to 45.31 kDa with an average of 30.7 kDa (Table 1). Two hundred thirty-one pair-wise sequence comparisons among the SsSWEETs showed that three pairs of SsSWEET13s shared the highest similarities in protein sequences ranging from 93 to 95%, and three pairs SsSWEET4s (a, b, and c) had the second highest protein sequence similarities ranging from 83 to 93%. The remaining 225 gene pairs had protein sequences identities ranging from 27 to 73% with an average of 39% (Additional file 2).

**Fig. 2:**
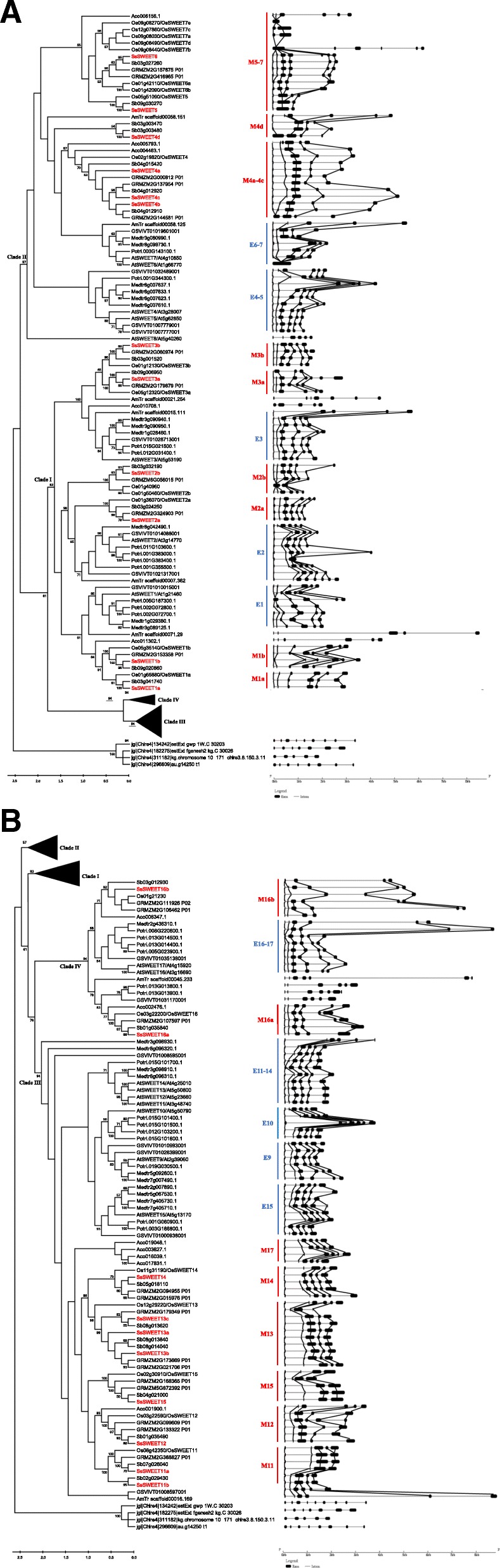
Phylogeny and schematic diagram for intron/exon organization of *SWEET* genes from 11 plant species. The corresponding exons with similar sequences based on sequence alignments were indicated with solid lines. **a** Clade I and Clade II. **b** Clade III and Clade IV

Comparisons of *SsSWEETs* with their orthologs in sorghum showed that two *SsSWEETs* (*SsSWEET3a* and *SsSWEET4d*) had larger protein sizes than their sorghum orthologs (Table 1). *SsSWEET3a* had one additional exon (the last exon) compared to its sorghum ortholog (*Sb09g006950*), whereas *SsSWEET4d* harbored a first exon larger than its ortholog (*Sb03g003470*) (Fig. 2). Deduced protein sequences comparisons showed that *S. spontaneum* and sorghum shared identities ranging from 33 to 98% with an average of 88%*. Saccharum* ESTs from the NCBI Genbank database were searched for *SsSWEETs* (Table 1).

To investigate the possible evolutionary functional constraints after the split of sorghum and *Saccharum*, the nonsynonymous to synonymous substitution ratio (Ka/Ks) was analyzed for orthologous gene pairs of *SWEETs* between sorghum and *S. spontaneum*. Apart from *SsSWEET4d*, the Ka/Ks ratios were less than 0.5, indicating that purifying selection was the dominant force for driving the evolution of *SsSWEET* genes (Table 2).

**Table 2:** Calculation of substitution rates of homologues *SWEET* genes between *Saccharum spontaneum* and *Sorghum bicolor*

	Gene pair		S-Sites	N-Sites	Ka	Ks	Ka/Ks
Clade I	*SsSWEET1*	*Sb09g020860*	105.1	644.9	0.0451	0.1673	0.2696
	*SsSWEET2*	*Sb03g032190*	106.2	646.8	0.0613	0.3314	0.1851
	*SsSWEET3a*	*Sb09g006950*	73.1	643.9	0.096	0.2051	0.4679
	*SsSWEET3b*	*Sb03g001520*	83.1	648.9	0.0413	0.1782	0.232
Clade II	*SsSWEET4d*	*Sb03g003470*	159.7	420.2	0.5298	0.5749	0.9215
	N/A	*Sb03g003480*	N/A	N/A	N/A	N/A	N/A
	*SsSWEET4a*	*Sb04g015420*	203.5	573.5	0.0086	0.3632	0.0238
	*SsSWEET4b*	*Sb04g012910*	135.9	614.1	0.0095	0.267	0.0355
	*SsSWEET4c*	*Sb04g012920*	140.9	372.1	0.0233	0.3578	0.0652
	*SsSWEET5*	*Sb09g030270*	113.5	654.5	0.0256	0.3868	0.0661
	*SsSWEET6*	*Sb03g027260*	92.8	528.2	0.02	0.4329	0.0461
Clade III	*SsSWEET11a*	*Sb07g026040*	164.6	744.4	0.008	0.1685	0.0475
	*SsSWEET11b*	*Sb02g029430*	109.1	676.9	0.0291	0.1804	0.161
	*SsSWEET12*	*Sb01g035490*	159.9	821.1	0.0186	0.1214	0.1535
	*SsSWEET13a*	*Sb08g014040*	148.2	775.8	0.0177	0.4333	0.0408
	*SsSWEET13b*	*Sb08g013840*	113.9	810.1	0.0484	0.4496	0.1077
	*SsSWEET13c*	*Sb08g013620*	108.8	707.2	0.0091	0.3827	0.0237
	*SsSWEET14*	*Sb05g018110*	142.2	763.8	0.0443	0.4067	0.1089
	*SsSWEET15*	*Sb04g021000*	161.9	741.1	0.0499	0.2817	0.1771
Clade IV	*SsSWEET16a*	*Sb01g035840*	109.8	844.2	0.037	0.2446	0.1512
	*SsSWEET16b*	*Sb03g012930*	102.9	386.1	0.0377	0.0916	0.4117

### Phylogeny and divergence of *SWEET* genes in *S. spontaneum* and angiosperms

To better understand the evolution of *SWEET* orthologs in different plants, we constructed an unrooted phylogenetic tree with 195 *SWEET* genes from 11 representative plant species using Maximum Likelihood (ML) and Neighbor-Joining (NJ) methods, respectively (Fig. 2, Additional file 3). The 11 representative plant species included dicots (*V. vinifera, A. thaliana, P trichocarpa* and *M. truncatula*), monocots (*A. comosus, O.sativa, Z. mays, S. bicolor and S. spontaneum*), *A. trichopoda* (basal angiosperms)*,* and *C. reinhardtii*. Consistently with a previous study [7], *SWEETs* from different species could be divided into four clades (I, II, III and IV) (Additional file 3). In the earliest diverging angiosperm, *A.trichopoda*, there were only 8 *SWEETs*, whereas in dicots and monocots the number of *SWEETs* varied between 12 and 25, indicating that gene expansion had occurred in *SWEET* genes in both monocot and dicot lineages (Fig. 1). Within the four clades, clade III was observed to have a great expansion of *SWEET* genes in both monocot and dicot lineages as highlighted by the evidence that there is only 1 in *A. trichopoda,* while there are at least 5 *SWEETs* in both monocots and dicots (Fig. 1).

Based on their phylogenetic distribution, angiosperm *SWEET* genes can be approximately divided into 27 subfamilies including 10 from dicots and 17 from monocots. These subfamilies are referred to as 10 eudicot subfamilies E1 (Eudicot1), E2, E3, E4–5, E6–7, E9, E10, E11–14, E15, and E16–17, and 17 monocot subfamilies, M1a (Monocot1), M1b, M2a, M2b, M3a, M3b, M4a-4c, M4d, M5–7, M11, M12, M13, M14, M15, M16a, M16b and M17 (Fig. 2). These eudicot subfamilies generally contained *SWEETs* from all four examined eudicot species (*V. vinifera, P. trichocarpa, A. thaliana,* and *M. truncatula)*, but had a different number among the eudicot species. Among all monocot subfamilies, only the M1, M4, M5–7, M12, and M16 subfamilies included *SWEET* genes from all five examined Poaceae species, indicating that the progenitors of those genes may have already existed prior to the split of Poaceae. Eight subfamilies, M2a, M2b, M3a, M3b, M11, M13, M14 and M15, existed in four monocotyledonous plant species, excluding *A. comosus*, thus demonstrating that these subfamilies originated before the pan-grass ρWGD event and after the rise of *Poales* from commelinids [43].

Recent duplications were observed to be frequent in *SWEETs* in both dicots and monocots. For example, *SWEETs* in *A. thaliana* within E4–5, E6–7, E16–17 and E11–14, *P. trichocarpa* within E1, E2, E3, E10, E15 and E16–17, *A. comosus* within M4a-4c and M17, *S. bicolor* in M4d, M11 and M13. These results suggested that *SWEET* families undergo active gene expansion in angiosperms.

We estimated the divergence time among four clades of *SsSWEET* gene family based on the pairwise synonymous substitution rates (Ks) in *S. spontaneum* (Table 3). The median values of pairwise Ks ranged from 3.483 to 3.682, corresponding to a divergence time ranging from 285.5 to 301.8 Mya, suggesting that the *SWEETs* in the four clades were ancient and divergent. Moreover, the divergence time among the three *SWEET13s* ranged from 19.7 to 22.1 Mya with an average of 20.1 Mya (Table 4). These results indicate that the *SsSWEET* family is an ancient gene family with recent gene duplication events in *Saccharum.*

**Table 3:** Divergence time among four Clade of *SsSWEET* gene family in *Saccharum spontaneum*

Clade	Median Ks	Gene pairs used	Divergence time (mya)
Clade I/Clade II	3.548	36	290.8
Clade I/Clade III	3.619	48	296.7
Clade I/Clade IV	3.682	12	301.8
Clade II/Clade III	3.483	48	285.5
Clade II/Clade IV	3.593	12	294.5
Clade III/Clade IV	3.610	16	295.9

**Table 4:** Divergence between paralogous *SsSWEET* gene pairs in *Saccharum spontaneum*

Gene1	Gene2	Ka	Ks	Ka/Ks	Divergence time (Mya)	*P*-Value(Fisher)
SsSWEET4a	SsSWEET4b	0.036	0.866	0.042	71.0	1.07E-28
SsSWEET4a	SsSWEET4c	0.093	0.968	0.096	79.4	7.20E-24
SsSWEET4b	SsSWEET4c	0.071	1.044	0.068	85.6	3.04E-21
SsSWEET13a	SsSWEET13b	0.020	0.269	0.076	22.1	1.39E-16
SsSWEET13a	SsSWEET13c	0.023	0.240	0.095	19.7	7.44E-14
SsSWEET13b	SsSWEET13c	0.021	0.250	0.085	20.5	1.04E-14

### Exon–intron organizations of *SsSWEET* genes

To investigate the structural characteristics and evolution of the *SWEET* gene family, we analyzed the pattern of intron/exon distribution and position of the *SWEET* genes, and then mapped them to the phylogenic tree (Fig. 2). The corresponding exons with similar sequences, based on sequence alignments, are indicated with a solid line in Fig. 2. The number of exons in the *SWEET* family of the examined plant species varied from one to eight, with more than half of the *SWEET* genes (107/195, 54.8%) having six exons, suggesting that the last common ancestor (LCA) of angiosperm *SWEETs* had six exons. Moreover, in the examined *SWEETs*, the exon sizes were conserved, whereas the variation in gene size was observed to be caused by intron insertions (Fig. 2).

### Conserved domains of SWEETs in *S. spontaneum*

The typical structure of plant SWEET proteins consists of seven predicted transmembrane (7-TM) helices forming two *MtN3_slv* domains (triple-helix bundles, THB) connected by a linker transmembrane helix (TM4) [44, 45]. We analyzed the domain architecture of SWEETs from *O. sativa, S. bicolor, Z. mays* and *S. spontaneum,* and found that 95.6% of SWEETs contain two *MtN3_slv* domains, indicating that maintenance of two domains is important for the function of SWEET proteins. However, several members harbor only one *MtN3_slv* domain, i.e. LOC_Os09g08270/OsSWEET7e, and LOC_Os01g40960, which may result in non-functionalization, neo-functionalization or sub-functionalization. Consistent with previous studies in plants [46, 47], all SWEET members harbored two *MtN3_slv* domains with an average of 291 amino acids in *S. spontaneum* (Additional file 4).

**Fig. 3:**
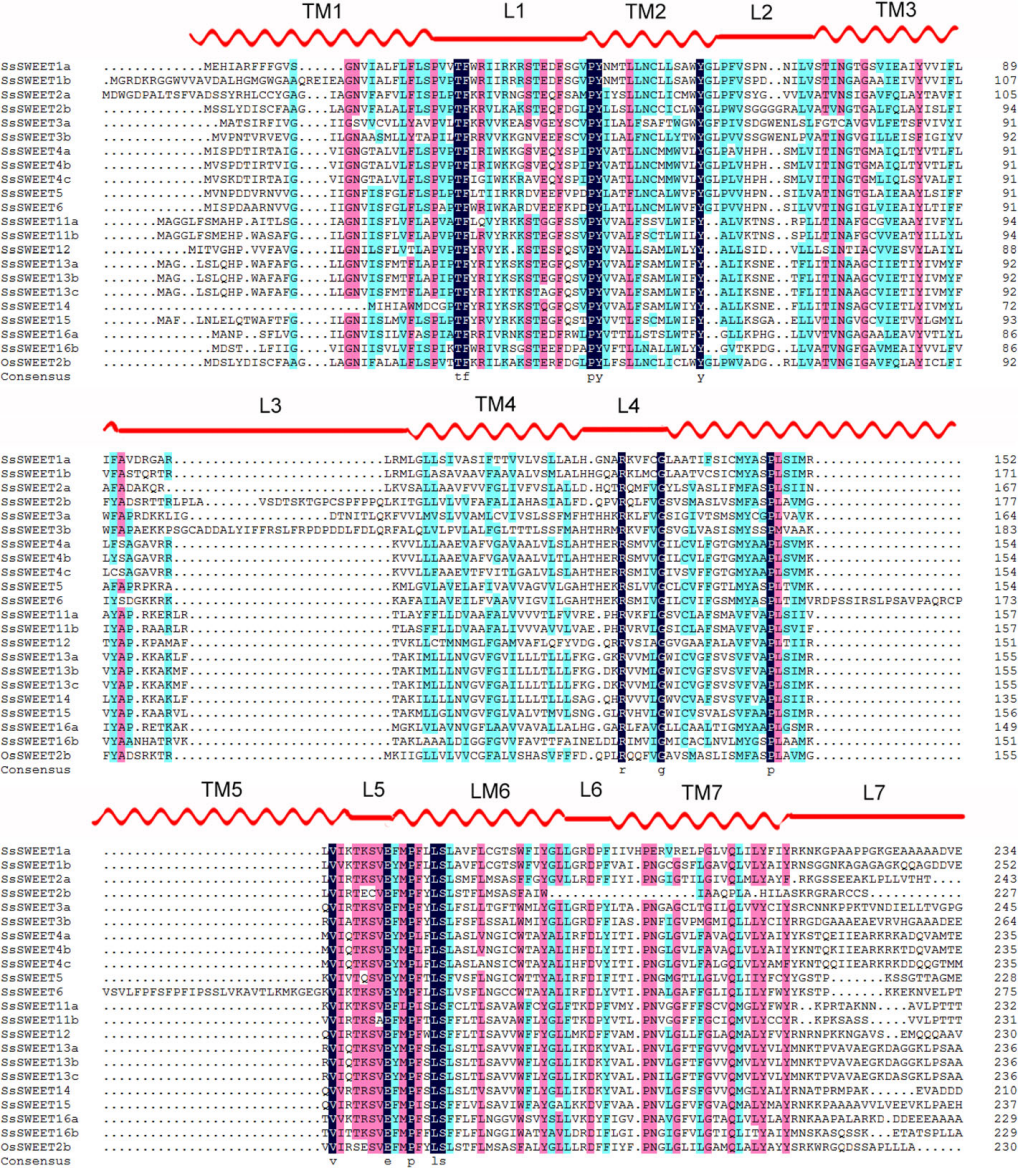
Sequence alignment of SsSWEETs and OsSWEET2b. Amino acid sequences of *SWEETs* were aligned using DNAman software. Predicted secondary structure assignment based on OsSWEET2b structure is indicated above the alignment

To further investigate the conserved status of the *MtN3_slv* domain within the gene family, 22 SWEET protein sequences from *S. spontaneum* and one rice protein OsSWEET2b sequence [48] were aligned to predict conserved domains (Fig. 3). The alignment showed that the residues T30, F31, P47, Y48, and Y61 on THB1 and R131, G136, P150, V157, E164, P167, L170, and S171 on THB2 were completely conserved in the 22 proteins in comparison with OsSWEET2b. In addition, more than 95% of the SsSWEET family members contained G16, P27, L52, N77, G80, S162, V163, Y184, D190, P196, N197, G200, Q207, Y211, and Y214 residues, suggesting that residues in the second *MtN3_slv* domain are more conserved than those in the first. Among them, the prolines in transmembrane 1(TM1), TM2, TM5 and TM6 were conserved in the 22 proteins, except for a cysteine-substitution in the TM1 of SsSWEET14, this being intuitive given the important roles of the proline tetrad in the transport mechanism [44]. In addition, two conserved residues (Asn77 and Asn197) surrounding the putative substrate-binding pocket in TM3 and TM7 [44], are both essential for AtSWEET1 activity at the corresponding positions (N73 and N192) [44, 49], and are retained in SsSWEET protein sequences except for SsSWEET2 and -3a.

### Expression analysis of *SWEET* genes in *S. spontaneum and S. officinarum*

To investigate the potential functions of *SWEET* genes in *Saccharum*, we compared the transcriptome expression profiles of all identified *Saccharum SWEET* genes using four experimental sets of RNA-seq datasets: 1) Different developmental stages 2) Leaf gradient 3) Diurnal cycles 4) Parenchyma and sclerenchyma cells. Expression levels of *SsSWEET2b* and *SsSWEET4b* were verified by reverse transcription quantitative PCR (RT-qPCR) in five tissue types from two *Saccharum* species, *S. officinarum* and *S. spontaneum* (Additional file 5)*.*

### Expression pattern of *SWEETs* at different developmental stages

**Fig. 4:**
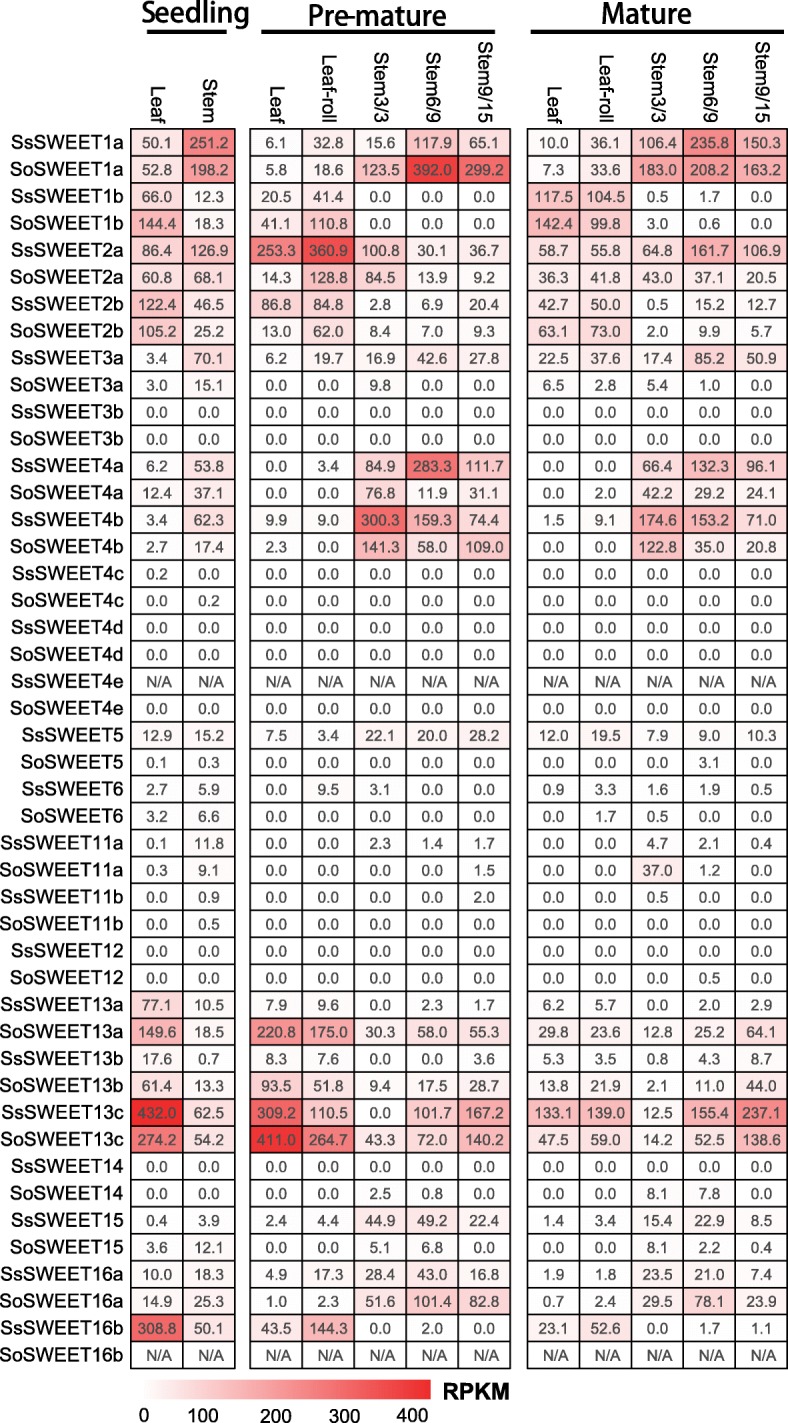
The expression pattern of *SWEETs* based on RPKM in different tissues of different stages in two *Saccharum* species

To explore gene functional divergence among the founding *Saccharum* species, we performed RNA-seq based comparative transcriptome profiling between two *Saccharum* species, *S. officinarum* and *S. spontaneum,* at different developmental stages and in five different tissues including the leaf (leaf roll and mature leaf) and 3 stalks (immature,, maturing and mature) (Fig. 4). Among the 22 *SWEET* analyzed genes, 8 genes (*SWEET3b*, *SWEET4c*, *SWEET4d*, *SWEET4e*, *SWEET6*, *SWEET11b*, *SWEET12* and *SWEET14*) were expressed at very low or undetectable levels in all examined tissues from the two *Saccharum* species. *SWEET3a, SWEET5* and *SWEET13a* were observed to have different expression levels in the two *Saccharum* species, *SWEET3a* and *SWEET5* having higher expression levels in *S. spontaneum* than in *S. officinarum*, whereas *SWEET13a* had much higher transcript levels in *S. officinarum* than *S. spontaneum*. Three *SWEET13s* (*13a*, *13b* and *13c*) displayed similar expression patterns with high expression levels, but *SsSWEET13c* showed the highest expression levels in all organs among the 22 analyzed genes. *SWEET1b* and *SWEET2b* also had high expression levels but displayed lower expression levels in mature stem tissues than in the other examined tissues from the two species. In contrast, *SWEET4a* and *SWEET16a* showed lower expression levels in the mature leaf and leaf roll than in the other tissues from both species.

### Expression patterns of *SWEETs* across leaf gradient segments

**Fig. 5:**
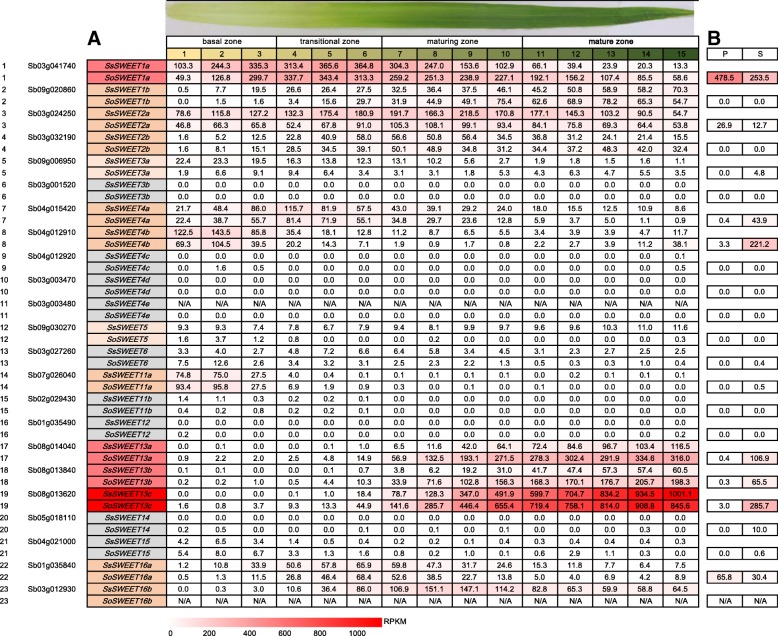
**a** The expression patterns of *SWEETs* based on RPKM across leaf gradients in two *Saccharum* species. **b** The expression level of *SWEETs* based on RPKM in Parenchyma cells and sclerenchyma cells from *S. officinarum*

To further investigate the functional divergence for sugar transport in the source tissues, we exploited the continuous developmental gradient of the leaf to profile the transcriptome of *SWEETs* in the two *Saccharum* species: the high sugar content species *S. officinarum,* and the stress tolerant species *S. spontaneum* (Fig. 5a). Similarly to the maize leaf [50], the *Saccharum* leaf can be divided into four zones: a basal zone (base, 1 cm above the leaf two ligules, sink tissue), a transitional zone (5 cm, 1 cm below the leaf one ligule, undergoing the sink-source transition), a maturing zone (10 cm, 4 cm above the leaf one ligule), and a mature zone (tip, 1 cm below the leaf two tip, fully differentiated and with active C_4_ photosynthetic zones). Consistently, 8 genes (*SWEET3b*, *SWEET4c*, *SWEET4d*, *SWEET4e*, *SWEET6*, *SWEET11b*, *SWEET12* and *SWEET14*), together with *SWEET5* and *SWEET15*, displayed undetectable levels, suggesting that these genes play a very small role in sugar transport in *Saccharum* leaves (Fig. 5a). *SWEET1a* showed higher expression levels in the transitional zone than in the other three zones of the leaf in both *Saccharum* species, whereas *SWEET2a* displayed higher expression levels in the transitional zone and maturing zone than in the other two zones in both *Saccharum* species. *SWEET1b*’s expression gradually increased from the base to the tip of the leaf of *S. sponteneum* but had higher levels of expression in the maturing zone than the other three zones of *S. officinarum*. The expression of *SWEET3a* gradually decreased from the base to the tip of the leaf in *S. sponteneum,* and showed higher levels in *S. sponteneum* than in *S. officinarum*. The overall expression levels of both *SWEET4a* and *SWEET4b* decreased from the leaf base to the tip in the two *Saccharum* species*.*

As with *SWEET3a*, *SWEET4b* presented higher expression levels in *S. sponteneum* than in *S. officinarum*. *SWEET11a* had much higher expression levels in the basal zone than in the other zones, suggesting that *SWEET11a* has an important role in sugar transport. Expression of three *SWEET13s* (a, b and c) dramatically increased in the maturing and mature zones of the leaf, indicating that these genes are involved in sugar transport for photosynthesis. *SWEET13c* had the highest expression levels among the *SWEETs* of the *Saccharum* species with higher expression in the maturing and mature zones than other zones of the leaf*.* Nevertheless, *SWEET16b,* which was undetectable in *S. officinarum,* showed higher levels in the maturing and mature zones than in the other examined zones of *S. sponteneum*.

### Expression pattern of *SWEETs* during the diurnal cycles

**Fig. 6:**
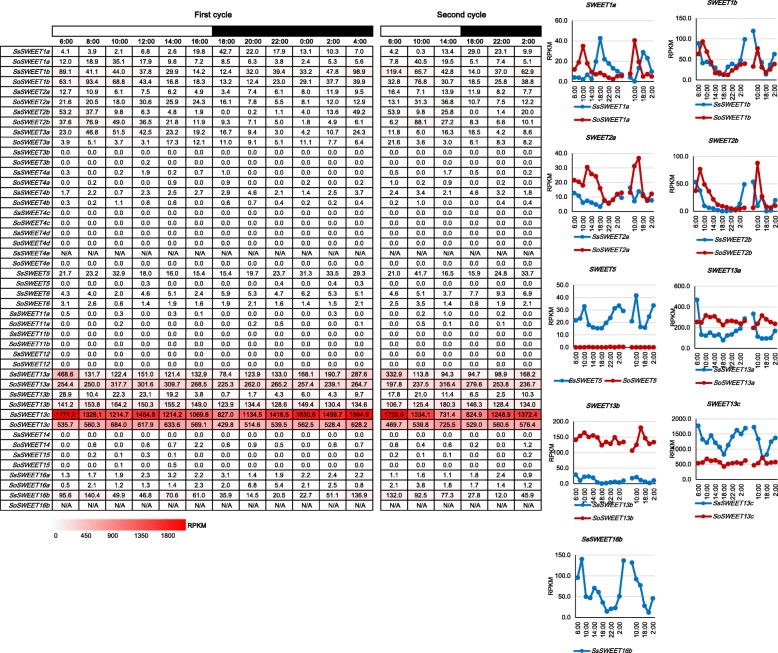
The expression pattern of *SWEETs* based on RPKM at different time periods

Many sugar-metabolizing enzymes, for example cell-wall invertases (CWINVs), vacuolar invertases (VINVs), and sucrose synthases are regulated by the circadian clock [51]. To investigate the expression patterns of *SWEETs* during the diurnal cycles, we collected samples for RNA-seq at 2-h intervals over a 24-h period, and at 4-h intervals over an additional 24-h from both *S. officinarum* and *S. spontanenum*. Consistently, 8 genes (*SWEET3b*, *SWEET4c*, *SWEET4d*, *SWEET4e SWEET6*, *SWEET11b*, *SWEET12* and *SWEET14*) were undetectable in the two *Saccharum* species, further supporting their limited role for sugar transport in *Saccharum* (Fig. 6). Moreover, *SWEET4a*, *SWEET4b*, *SWEET11a, SWEET15,* and *SWEET16a* also showed very low expression levels over the two 24-h periods. 8 *SWEETs* (*SWEET1a*, *SWEET1b*, *SWEET2a*, *SWEET2b*, *SWEET5*, *SWEET13a*, *SWEET13b, SWEET13c* and *SWEET16b)* were observed to have different diurnal expression patterns in the two *Saccharum* species, with *SWEET1a* having a peak expression by the late afternoon in *S. spontaneum*, while *S. officinarum* peaked in the morning. Except for *SWEET1a*, the remaining 7 genes had peak expression levels in *S. spontaneum* in the morning. In *S. officinarum, SWEET1b* and *SWEET2b* had a peak in expression in the morning, whereas *SWEET13a, SWEET13b, SWEET13c,* and *SWEET2a* had a peak expression at noon, suggesting *SWEETs* correlated with diurnal rhythms in both *S. spontaneum* and *S. officinarum*, and that the difference in gene expression observed during the circadian rhythm may be related to carbohydrates metabolism*.*

### Expression levels of *SWEETs* in parenchyma and sclerenchyma cells from the high sugar content *S. officinarum*

Parenchyma cells in mature sugarcane stalks can accumulate sugars to levels with an osmotic potential of − 2.2 MPa [52]. To further investigate the functional divergence for sugar accumulation, we compared the transcriptome profiles of parenchyma cells and sclerenchyma cells separated from mature sugarcane stalk in *S. officinarum* (Fig. 5b)*.* The expression levels of *SWEETs* varied between the two cell types. *SoSWEET1a* had higher expression levels than the other 21 *SWEET* genes, and together with *SoSWEET2a* and *SoSWEET16a* had higher expression levels in parenchyma than in sclerenchyma cells, indicating that these genes may be involved in the accumulation of sugars in parenchyma cells. In contrast, the expression of five genes (*SoSWEET4a*, *SoSWEET4b*, *SoSWEET13a*, *SoSWEET13b* and *SoSWEET13c*) in sclerenchyma cells was significantly higher than that in parenchyma cells, indicating that these genes play a role in sugar transport in *Saccharum*.

### Expression of *SWEET16b* was undetectable in *S. officinarum*

*SWEET16b* was expressed in *S. spontaneum* but undetectable from the transcript of *S. officinarum,* indicating the absence of this *SWEET* gene in *S. officinarum.* Genomic DNA was analyzed by polymerase chain reaction (PCR) and the results confirmed that *SWEET16b* was lost in *S. officinarum* (Additional file 6).

## Discussion

The *SWEET* family of sugar transporters has been recently identified in higher plants [7] . *SWEETs* also play a key role in pathogen susceptibility related to their function in sugar transport. Therefore, it is understandable that the *SWEET* gene family is the center of interest in the study of sugar accumulation and disease resistance in sugarcane. However, in spite of the great advantages offered by genome sequencing technologies in the last decade, the sugarcane genome remains unavailable due to its complex genetic background. Here, we identified 22 full-length *SWEET* genes from *S. sponteaneum.* Phylogenetic trees were made to study the evolution of *SWEETs* in sugarcane and other representative plants. Furthermore, based on RNA-seq data, *Saccharum SWEET* genes displayed specific temporal and spatial expression patterns, which provided the clues for investigating their specific functions.

### Evolution of the *SWEET* family in *Saccharum* and angiosperms

The occurrence of WGDs, or polyploidy, is thought to be a common driving force for the evolution of angiosperms, the most recently evolved and most successful lineage of land plants [53–55]. These WGDs, which are associated with the origin of the recent common ancestor of extant angiosperms (ε), pan-core eudicots (γ), and monocots (ρ), have been revealed by integrated synteny, age estimates of gene duplication, and phylogenomic analysis [53, 55]. Recent studies revealed that pineapple has one fewer ancient WGD (ρ) event than the other sequenced grass genomes. This recently available WGD information, together with the 11 plant species representing major WGD events in angiosperm, made it possible to study *SWEETs* gene evolution in angiosperms. Consistently with previous studies [23, 42, 56, 57], our results showed that SWEETs from different species could be divided into four clades in duplicated descending order: clade II, clade I, clade III and clade IV. Based on the estimate of divergence in paralogous *SWEET* genes in pear, plant *SWEET* genes were predicted to have emerged in the Neoproterozoic period [57], which was much earlier than the origin of angiosperms. Thus, *SWEETs* were deduced to have multiple last common ancestors in angiosperms. In this study, based on estimation of the divergence time among four clades of *SsSWEET* gene family, the *SsSWEET* families originated (about 285.5 to 301.8 Mya, Table 3) before ε WGD in angiosperms (about 200Mya) and after ζ WGD in seed plants (about 330Mya) [53]. Therefore, the last common ancestors of the four clades in angiosperms were deduced to be retained from the ζ WGD.

In clade I, three groups, M3a/M3b/E3, M2a/M2b/E2, and M1a/M1b/E1 could be assumed to have originated from three LCAs in angiosperms, which was supported by the evidence that each of the three groups contained the genes from all the representative plants (Fig. 2). One hypothesis is that the LCAs of M2a/M2b/E2 and M1a/M1b/E1 originated from the ε WGD event after these two groups split from the LCA of clade I. The ρ duplication occurred before the origin of the lineages leading to rice, wheat and maize but after separation of the lineages leading to the grasses and pineapple 95–115 million years ago [58, 59]. Therefore, we can assume that M1a/M1b originated from the ρ duplication as each of the pair of group genes were observed to originate from a duplication event after the divergence of the lineages leading to the grasses and pineapple. In contrast, M2b may have diverged from M2a before the origin of angiosperms as M2b was separated from the subclade M2a/E2, which contained all the representative plant species. The group of M3a/M3b/E3 contained two genes (*AmTr-scaffold00021.254 and AmTr-scaffold00015.111*) from *A. trichopoda*, with one clustered with monocot genes and another clustered with dicot genes, which is explainable given that the sister of all other extant angiosperms, *A. trichopoda*, underwent gene duplication before the separation of dicots and monocots.

Furthermore, either monocots or dicots were subjected to gene loss in the early stages of their splits. In contrast, the expansions of *SWEETs* in dicots in this clade were caused by the recent gene duplication event rather than the ancient WGD event. This is demonstrated by Arabidopsis having two additional WGDs (α and β) but not containing additional *SWEET* members. In addition, *SWEET* genes from the same plant species were phylogenetically distributed together (for example, *SWEET* genes from *P. trichocarpa* within E1 and E2, *SWEET* genes from *M. truncatula* within E3). Based on the phylogenetic analysis**,** the evolutionary history of *SWEETs* in *Saccharum* can be sorted by age in duplicated descending order: *SsSWEET2b*, *SsSWEET2a*, *SsSWEET3b*/*SsSWEET3a* and *SsSWEET1a*/ *SsSWEET1b*.

In clade II, *SWEETs* expansion in *Poales* were also assumed to be mainly caused by ρ WGD. M5–7 groups have two subgroups of genes, of which one clustered with one pineapple *SWEET*, whereas the recent gene duplications contributed to *SWEET* expansion in rice. In M4a-4c groups, *Poales* plants likely retained the *SWEETs* inherited from σ WGD (for example: *Aco005793.1* and *Aco004463.1*), and one of the ancestors (the common ancestor of *SsSWEET4b/4c)* underwent ρ WGD in the lineages leading to rice, sorghum and maize and sugarcane which generated two SWEETs (for example: *SsSWEET4b*/*4c*). M4d was only found in the *Trib. Andropogoneae*, making it possible to hypothesize that this group of genes originated within the *Trib. Andropogoneae*. Moreover, the divergence times of *SsSWEET4a*/*b*/*c* were estimated to range from 71 to 85 Mya (Table 4), which are consistent with the occurrence of the ρ WGD event in the cereal lineage about 70 million years ago [60, 61], supporting the hypothesis *SWEETs* expansion in *Poales* was mainly caused by ρ WGD.

Similarly to clade I, in dicots, recent duplications (tandem duplication) were the main driving force for *SWEET* expansion. Specifically for example, *SWEETs* from *V. vinifera* within E4–5, and *SWEETs* from *M. truncatula* within E6–7. Therefore, we can assume that the age in duplicated descending order for *SWEETs* in *S. spontaneum*is: *SsSWEET4d*, *SsSWEET4a*, *SsSWEET4b*/*SsSWEET4c*/*SsSWEET5*/ *SsSWEET6*.

Synteny could have been lost after chromosome rearrangements, fusion, and fractionation in the plant genome, complicating the phylogenetic analysis for gene evolution. In clade III, M12 containing *SWEETs* from the four *Poales* plants indicated that *SWEETs* were inherited from σ WGD. M11, M13, M14 and M15 were suggested to have originated from the lineages leading to rice, sorghum and maize as pineapple *SWEETs* were absent in these groups.One subgroup (containing *Sb02029430* and *SsSWEET11b*) in M11 was indicated to have originated from its sister subgroup in a recent duplication in *Trib. Andropogoneae*. Subgroups M11/M12/M15 and M13/M14 were assumed to have originated from an LCA after the split of dicots and monocots. Subgroups M13 contained the recently duplicated SWEETs in *Panicoideae*. The estimated divergence times of *SsSWEET13a*/*b*/*c* in M13 ranged from 19.7 to 22.1 Mya (Table 3), which were similar to the divergence time between maize and *Trib. Andropogoneae* (sorghum) about 20 Mya [62]*.* Therefore, these *SWEETs* of M13 in *Panicoideae* were duplicated after the rise of *Panicoideae* in *Gramineae*. Similarly, *SWEET* expansion in dicots was mainly attributed to the recent duplications, especially the tandem duplication. Based on this deduction, we can speculate that the age in duplicated descending order for *S. spontaneum* SWEETs is: *SsSWEET15*, *SsSWEET12*, *SsSWEET11a*, *SsSWEET11b* for the group M11/M12/M15, and *SsSWEET14*, *SsSWEET13c*, *SsSWEET13a*/*SsSWEET13b* for the group M13/M14.

Clade IV contained the fewest SWEET genes among the four groups. *SWEETs* in both M16a and M16b were indicated to be inherited from σ WGD as both of these groups contained *SWEETs* from the four *Poales* plants. Furthermore, consistent with the other three groups, *SWEET* expansion in dicots was mainly attributed to the recent single gene duplications. In *S. spontaneum,* both *SsSWEET16b* and *SsSWEET16a* were clustered with the five examinated monocot species including pineapple*,* thus were predicted to have originated before the rise of *Poales* from commelinids and not generated by the ρ WGD. Moreover, *SsSWEET16a* was suggested to have been inherited from the LCA of angiosperms.

Gene families were retained from WGDs or single-gene duplicates during the evolutionary process. Among the single-gene duplications, transposed duplicates were suggested to evolve faster than tandem or proximal duplicates [63–66]. Moreover, single-gene duplicates have higher levels of expression divergence [64–67], functional innovation [68, 69], network rewiring [69] and epigenetic changes [65] than duplicated genes retained from WGDs. Based on these theories, monocot *SWEET* genes may in general have higher levels of gene functional divergence than dicot *SWEET* genes.

In parallel to phylogenetic distributions, the structure of *SsSWEET* genes underwent substantial variation during evolution, with the number of introns varying from one to eight. For eudicots, the finding that most (82.5%) *SWEETs* are six-exon genes led us to speculate that a basic gene model consisting of six exons and five introns could be deduced in the ancestral eudicot *SWEET* genes, from which all gene structures can evolve by the insertion and / or loss of introns. However, the exon/intron organization in monocot *SWEET* genes is more diverse than in dicots, which may be because *SWEETs* in monocot were mainly retained from WGDs, while *SWEETs* in dicots mainly originated from the recent single-gene duplicates. Given the fact that 20.0%, 30.9% and 40.2% of *SWEET* genes from monocots contain four, five and six exons, respectively, it is reasonable to postulate that apart from the six-exon ancestral genes of many clades (such as M1a, M1b, M5–7 etc.), four-exon and five-exon genes are the next ancestral genes since they are present in the ancestor of *Poaceae* plants, for example, the ancestral genes of subfamily M15, M14 and M11.. More unexpectedly, all members in M14 harbor 6 exons except for *OsSWEET14* and *SsSWEET14,* which contain five exons. In contrast, its sister clade M13 was composed of five-exon genes except the six-exon *LOC_Os12g29220/OsSWEET13* and *GRMZM2G179349_P01* (4 exons), suggesting that the gain of an intron or fusion of exons have occurred in the ancestor of the subfamilies M14 and M13, respectively.

Typically, SWEETs contain seven transmembrane domains (TMs) consisting of two tandem repeats of 3-TM units separated by a single TM unit [44, 45], but ExtraSWEET proteins with more TMs have been reported in *Vitis vinifera* [70]. In this study, the 22 identified SsSWEETs possess 6–8 TMs, with a large proportion of SsSWEETs (68%) containing seven TMs (Table 1). All *SWEET* members contained two *MtN3_slv* domains, implying that SWEET proteins in *S. spontaneum* were generated by the duplication and fusion of a SemiSWEET [45]. The pI values of the SsSWEETs ranged from 5.41 (SsSWEET12) to 9.57 (SsSWEET13a), with the majority of members (18) exhibiting pI values > 7(Table 1). The pI values of the remaining four (SsSWEET4d, SsSWEET12, SsSWEET15 and SsSWEET16b) were lower than 7, and were distributed in clade II (SsSWEET4d), clade III (SsSWEET12 and SsSWEET15), and clade IV (SsSWEET16b), suggesting the independence between pI divergence and gene evolution.

### Gene expression and functional divergence of *SWEETs* in *Saccharum*

Gene expression patterns are highly correlated with gene function in plants [71]. Previous studies on the *SWEET* sucrose/hexose efflux transporter genes have shown that they may play multiple roles during plant development based on their transport activity in the heterologous expression system and expression patterns in some plant species [7, 8, 45, 72]. In this study, with the aim of understanding the potential functions of *SWEET* in *Saccharum* species, we investigated *SWEET* gene expression patterns based on four sets of RNAseq data.

*SWEET1s*: *SWEET1a* has higher expression levels in the transitional zone and maturing zone than in the other analyzed zones, and is the dominant gene expressed in these two zones in the developmental gradient leaf sections, indicating that *SWEETs* have complementary gene expression for transporting sugar in different leaf sections. *SWEET1a* and *SWEET13s* were suggested to have functional specificity in the different leaf sections as *SWEET13s* (mostly is *SWEET13c*) displayed a dominant expression pattern in highly photosynthetic zones, while *SWEET1a* was predominantly expressed in the low photosynthetic zones. *SWEET1a* was also observed to be mainly expressed in the stem and had higher expression levels in the stem of *S. officinarum* than that of *S. spontaneum* at the premature stage. In many plants, sugar accumulation in meristematic sinks is source-limited and is sink-limited in storage sinks [73]. *SWEET1a* is also expressed at high levels in the sclerenchyma and parenchyma cells from the mature stalk (RPKM> = 250). It is possible that *SWEET1a* contributed to breaking through the limitations of the storage sink, and thus gave rise to the high sugar content in *S. officinarum*. In contrast, *SWEET1b* was mainly expressed in the leaf tissue and the mature zone of the leaf in the two *Saccharum* species, and displayed peak expression levels in the morning, while it was undetectable in both sclerenchyma cells and parenchyma cells of the mature stalk in *S. officinarum*. Therefore, *SWEET1b* appeared to be a sucrose starvation-induced gene involved in sugar transport in the highly photosynthetic zones in *Saccharum*.

*SWEET2s*: Similarly to *SWEET1a*, both *SWEET2s* were more abundant in the middle zone than in the other zones of the leaf sections, and were expressed at higher levels in *S. spontaneum* than in *S. officinarum*. *SWEET2a* was also observed to be upregulated in *S. spontaneum* in comparison to *S. officinarum* in the leaf tissue, especially at the maturing stage, but had lower expression levels in the leaf than in the stem at the mature stage in *S. spontaneum*. In sugarcane, the leaf photosynthetic activity decreased significantly during maturation due to senescence and negative feedback from the increased culm sucrose content [74–77]. Thus, we speculated that *SWEET2a* is involved in regulating source capacity and sink accumulation during the maturing stage in *S. spontaneum. SWEET2b* was mainly expressed in the leaf tissues in the two *Saccharum*, similarly to its orthologs in sorghum [78], indicating that this gene may be involved in sugar transport within the source tissues.

*SWEET4s*: *SWEET4c* have been well characterized in maize [19]. *SWEET4* genes have three copies (*SWEET4a*, *SWEET4b* and *SWEET4c*) in *Saccharum*, sorghum and maize, and only one copy in rice (*Oryza sativa*), suggesting that three paralogs in the three *Panicoideae* species evolved from an LCA of rice and the *Panicoideae* species. Moreover, both *ZmSWEET4c* and *OsSWEET4* were revealed to be responsible for transporting sugar into the endosperm and contributing to seed filling [19], suggesting a conserved function for *SWEET* after the divergence of maize and rice. In contrast to maize, in the two *Saccharum* species, *SWEET4c* displayed very low expression levels in all tissues, while the other two paralogs (*SsSWEET4a, −4b)* with higher expression levels than *SWEET4c* were mainly expressed in stems of seedlings and mature plants. This led us to speculate that *SWEET4* genes may also be specifically related to hexose transport across the basal endosperm transfer layer, and contribute to sink strength in sugarcane, but *SWEET4c* may not be the pivotal player among the three *SWEET4s* for this reproductive process in *Saccharum*. *SWEET4d* was undetectable in the examined tissues, and similarly, its ortholog (*Sb03003480*) showed no expression in sorghum [78]. *SWEET4d* was assumed to have originated after the split of sorghum and *Saccharum* based on the evolutionary analysis herein before, adding further support that the *SWEET4d* lineage in *Trib. Andropogoneae* is functionally redundant. Furthermore, the absence of another orthologous tandem duplication gene (*Sb03g003480*) in the *Saccharum* species was assumed to be caused by gene fusion after the divergence of sorghum and *Saccharum*, together with a high divergence of sequence similarity of *SWEET4d*/*Sb03g003470* (33%), suggesting that the *SWEETs* in M4d were potentially functionally redundant in *Trib*. *Andropogoneae*. The Ka/Ks of *SWEET4d*/*Sb03g003470* was 0.92, which adds further support to the hypothesis that *SWEETs* in M4d are potentially functionally redundant in *Trib. Andropogoneae.*

*SWEET11s*: *SWEET11a* has a higher expression level in the basal zone of the leaf, and its ortholog (*Sb07g026040*) was specifically expressed in the panicle in sorghum [78]. In sugarcane, *SWEET11a* may coordinate the delivery of sucrose from the leaf with the needs of the development of panicle. *SWEET11b* displayed very low expression levels in the examined tissues in the two *Saccharum* species, and its ortholog was also specifically expressed in the panicle in sorghum [78]. *OsSWEET11*, the close ortholog of both *SWEET11a* and *SWEET 11b*, together with five phylogenetically close *OsSWEETs* were revealed to confer TAL effector-mediated susceptibility to *Xanthomonas* in rice [22]. Therefore, we hypothesized that the two *SWEET11s* were the candidates which may mediate the infection of the pathogen in *Saccharum.*

*SWEET13s: SWEET13s* were recently duplicated genes. Three paralogues *SWEET13a, −13b,* and *-13c* showed similar expression patterns in the examined tissues, where *SWEET13c* was highly expressed in all the examined tissues including leaves and stems. Consistently, *SWEET13c* (*Sb08g013620*) had the highest expression among the *SWEET* families in sorghum [78]. *SWEET13c* appeared to be the pivotal player among the *SWEETs* for sugar accumulation in *Saccharum* when expressed in mature leaves and stalks*. SWEET13a* had a diurnal peak expression in the morning in *S. spontaneum*, but in *S. officinarum SWEET13a* had its diurnal peak expression was at noon. This can be explained as *SWEET13a* is a sucrose starvation-induced gene since depletion of nocturnal reserves leads to the activation of sucrose in the low sugar content in *S. spontaneum*. In contrast to *SWEET1a*, the expression of *SWEET13s* dramatically increased from the maturing to mature zones, indicating that *SWEET13s* are mainly involved in photosynthesis for sugar transport. Therefore, we hypothesized that *SWEET13s* might play a role in removing the photo assimilated sugar from source leaves, thus minimizing sugar repression of photosynthesis. In maize, SWEET13s were suggested to also serve as potential key transporters in the evolution of C4 photosynthesis [79], adding further support that SWEET13s are key players for the sugar efflux of sugar transportation in photosynthetic tissues. Furthermore, *SWEET13s* were mainly expressed in sclerenchyma cells of mature stems in *S. officinarum* (Fig. 5b), and both *SWEET13a* and *SWEET13b* have higher expression levels in *S. officinarum* than in *S. spontaneum*. Consequently, we considered *SWEET13c* as a strong candidate for sucrose transport from source to sink, while *SWEET13a* and *SWEET13b* may contribute to the sugar content difference between *S. officinarum* and *S. spontaneum*.

The SWEET family members in clade III were revealed to transport sucrose and small amounts of fructose [7, 23, 56, 80]. In *Saccharum*, at least 6 sucrose transporters (SUTs) were identified, and SUT1 was dominantly expressed among the families [34, 81]. SUT1 has been shown to have high expression levels in premature internodes, and decreased expression levels in mature internodes in *Saccharum hybrid* and *S.officinarum* [34, 81]. SUT1’s expression increased with stem maturation in *Saccharum* and had higher gene expression in *S. officinarum* than in *S. spontaneum*. SUT1 was also demonstrated to be highly selective for sucrose and to function in loading sucrose from the vascular tissue into the stem parenchyma cells [81, 82]. It is possible that *13a, 13b,* and *13c* cooperate with SUT1 for sucrose transport. Subsequently, SUTs load sucrose into the phloem of leaf minor veins, and also function to retrieve sucrose from the apoplast during transport, sucrose is concentrated in the sieve element–companion cell complex [78].

*SWEET16s*: *SWEET16a* presented higher expression levels in the stem tissues than in other tissues of the two *Saccharum* species, and had two times the level of transcripts in parenchyma cells compared to sclerenchyma cells (Fig. 5b). In sorghum, the ortholog of *SWEET16a* (*Sb01g035840*) was mainly expressed in the panicle [78]. In Arabidopsis, the close orthologs *AtSWEET16* and *AtSWEET17* distributed in clade IV, localize to the tonoplast, and *AtSWEET17* is a key determinant of leaf fructose content [21]. *SWEET16b*’s expression was not detected in *S. officinarum*, but displayed rhythmic diurnal expression patterns with higher expression levels in the early developmental compared to mature developmental stages in the leaf tissues of *S. spontaneum*. These results led to the speculation that *SWEET16a* may be involved in the sugar accumulation in the sink tissues of *Saccharum*, and *SWEET16b* may contribute to the sugar content divergence between the two *Saccharum* species.

In this study, 8 genes (*SWEET3b*, *SWEET4c*, *SWEET4d*, *SWEET4e*, *SWEET6*, *SWEET11b*, *SWEET12* and *SWEET14*) had very low or undetectable levels of expression in all the examined tissues from the two *Saccharum* species. These results led us to deduce that these 8 genes have insignificant roles for sugar accumulation in *Saccharum*. However, four of the eight orthologs, *SWEET4c*, *SWEET6*, *SWEET11b* and *SWEET14*, were expressed in the panicle tissues in sorghum [78], indicating that these 4 genes take part in sugar transport for reproductive rather than vegetative growth. Two of the rice orthologs, *SWEET3b* and *SWEET12* were induced by arbuscular mycorrhizal fungi (AMF) symbiosis in the root tissues [83], indicating that *SWEET3b* and *SWEET12* could have potential functions in creating symbiotic relationships at the root interface.

**Fig. 7:**
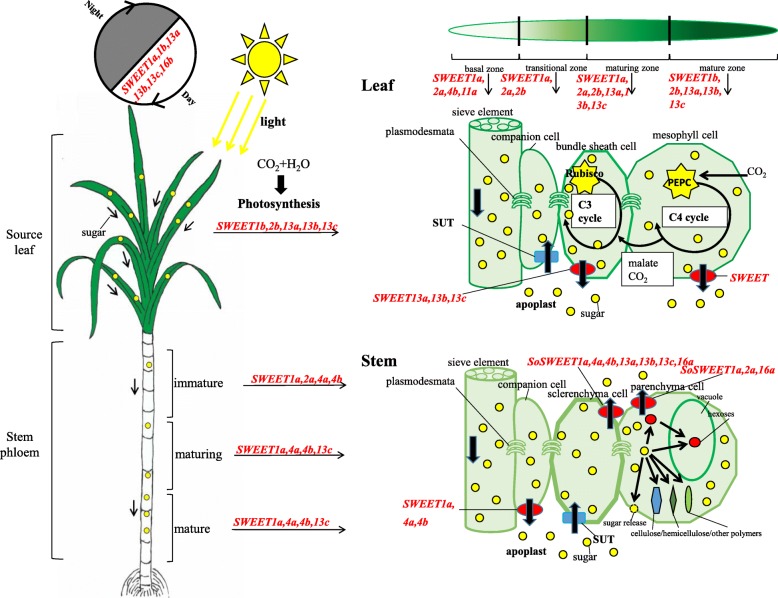
Schematic models for roles of SWEET proteins in phloem loading and unloading based on gene expression profiles in sugarcane. Notes: In the leave tissues, *SWEET13a/13b/13c* were the dominant expressional genes in mature and maturing zones, *SWEET1b* was expressed predominantly in the mature zone, *SWEET2b* was expressed in the transitional zone and the maturing zone, *SWEET1a/2a* were expressed in the basal zone (sink tissues), transitional zone and maturing zone, and *SWEET4b/11a* were expressed in the basal zone (sink tissues). These observations suggested that *SWEET1b/2b/13a/13b/13c* are associated with sucrose efflux in the photosynthetic tissues, while, *SWEET1a/4b/11a* are associated with sucrose unloading from the leaf to the stem. Sucrose is synthesized by the Calvin cycle from carbon dioxide and water in mesophyll cells. *SWEET13a/b/c* was the most abundantly expressed gene in the maturing and mature zones of leaves, and was suggested to play a role in the efflux of photosynthesized sucrose to the leaf apoplast on the basis of the function of its homologs in *Arabidopsis* (*AtSWEET11* and *AtSWEET12*) [95]. Moreover, *SWEET1b/13a/13b* presented a diurnal expression pattern, indicating that these three genes are regulated by sunlight. In the stem tissues, *SWEET1a/4a/4b* were constitutively expressed in the whole stalk, *SWEET2a* was mainly expressed in the immature stem, *SWEET13c* was expressed in the maturing and mature stem, suggesting that *SWEET1a/4a/4b* are involved in sucrose unloading and accumulation in sink tissues, whereas *SWEET2a* is associated with sucrose efflux from the source to the sink tissues, and *SWEET13c* is likely to be involved in sugar accumulation in the mature stem. Synthesized sucrose is transported from the leaf through sieve elements and accumulates in the parenchyma cells of sugarcane stalks. Therefore, *SoSWEET2a* may be involved in the accumulation of sugars in parenchyma cells and sugar transporting in sclerenchyma cells. *SoSWEET4a*, *SoSWEET4b*, *SoSWEET13c* may contribute to the sugar transport in sclerenchyma cells. *SoSWEET16a* had higher expression in parenchyma cells than in sclerenchyma cells, indicating that these genes may be involved in the accumulation of sugars in parenchyma. Reference: Chen LQ, Cheung LS, Feng L, Tanner W, Frommer WB. Transport of sugars. Annu Rev. Biochem. 2015; 84(84):865–94

Synthesized sucrose is transported from the leaf through the sieve elements and accumulates in the parenchyma cells of sugarcane stalks. Based on the expression patterns of these 22 *SsSWEET* genes, we produced a model (Fig. 7) illustrating the spatial and temporal expression of these genes in sugarcane plant tissues and cells. In the photosynthetic leaf tissue, specifically in the maturing zones of the leaf, *SWEET1b, 2b, 13a, 13b,* and *13c* are the predominant players in efflux of photosynthesized sucrose to the leaf apoplast and eventually to the sink. Moreover, *SWEET1b/13a/13b* presented rhythmic diurnal expression patterns, indicating that the three genes are regulated by sunlight. In the maturing zone of the leaf, *SWEET1a and 2a* served the same role as *SWEET1b* in the mature zone for sugar transportation. In the transition zone of the leaf, where the photosynthesis is much less active than the maturing and mature zones, *SWEET1a, 2a,* and *2b* are responsible for sugar transport. The basal zone acts as the immediate sink tissue and here *SWEET1a, 2a, 4b,* and *11a* play major roles in the accommodation of the products of photosynthesis and the unloading of these products from the leaves to stems. *SWEET1a*, *4a*, and *4b* are constitutively expressed in the whole stalk, thus suggesting their involvement in sucrose transport from companion cells to the apoplast and to parenchyma cells for the complete process of sucrose accumulation. *SWEET2a* and *SWEET13c* were mainly expressed in the immature and maturing/mature stem, respectively, suggesting their tissue-specific roles in sucrose efflux from the source to the sink tissues. *SWEET2a* may be involved in sugar accumulation in parenchyma cells and sugar transport in sclerenchyma cells. *SWEET4a, SWEET4b, SWEET13c* may contribute to sugar transport in sclerenchyma cells mostly for cellulose synthesis.

## Conclusions

In this study, we identified 22 *SWEET* genes from *S. spontaneum*. Phylogenetic analyses based on orthologs from 11 representative plant species revealed four clades which could subsequently be sorted by age in duplicated descending order: clade II, clade I, clade III and clade IV. The expansions of SWEET genes were mainly caused by recent gene duplications in dicot plants, while SWEETs were mainly retained from the ancient WGD in monocot plant species. In *Saccharum*, *SWEET16a*/*16b* were retained from ε WGD; three gene pairs, *SWEET1a*/*1b*, *SWEET3a*/*3b* and *SWEET5*/*6* were assumed to be retained from ρ WGD; two subgroups, *SWEET13a*/*13b*/*13c*/*14* and *SWEET11a*/*11b*/*12*/*15,* originated from ρ WGD, and subsequently *SWEET13a*/*13b*/*13c* were generated by tandem duplicates, while *SWEET11b* was recently duplicated from *SWEET11a* in *Trib. Andropogoneae*; and *SWEET4d* originated with the rise of *Trib. Andropogoneae*. Gene expression patterns and ortholog comparisons provided the clues to understand the potential function of the *SWEET* families in *Saccharum*. We established that *SWEET1b* was a sucrose starvation-induced gene involved in sugar transport in highly photosynthetic zones; *SWEET13c* was the key player in the efflux of sugar transportation in the mature photosynthesis tissues, *SWEET4a* and *SWEET4b* were mainly involved in sugar transport in the stalk. *SWEET1a*\*2a*\*4a4b*\*13a*\*16b* appeared to be genes contributing to the differences in sugar content between *S. spontaneum* and *S. officinarum*. Despite the large amounts of gene expression data for SWEETs, it remains unknown how *SWEETs* are coordinated with other sugar transporter genes for sugar metabolism, and how they support the accumulation of sugars in *Saccharum*. These results offer a useful basis for future research aiming to understand the physiological roles of SWEET gene and molecular mechanisms of sucrose metabolism in sugarcane. Experiments such as yeast-one-hybrid screens, characterization and dissection of the spatio-temporal expression, chromosome immune precipitation (ChIP) assays, yeast complementation and uptake assays and gene editing technology like CRISPR-Cas9 system, could be performed to elucidate the function of these genes.

## Materials and methods

### Plant materials

Two *Saccharum* species, LA-Purple (*S. officinarum*, 2n = 8x = 80, originated in USA) and SES-208 (*S. spontaneum*, 2n = 8x = 64, originated in USA) were used in this study [33].

For the different developmental stages experiment: Tissue samples were obtained from leaf roll, leaf, top immature internode (i.e. Stem3), premature internode (i.e. Stem 9 for LA-Purple and Stem 6 for SES-208) and mature internode (i.e. Stem 15 for LA-Purple and Stem 9 for SES-208) as previously described [34, 35].

For the leaf gradient experiment:*Saccharum* plants were grown with lamps at light intensity of 350 μmol/m2/sec, 14:10 l/D, 30 °C L/22 °C D and 60% relative humidity. Tissue was collected after planting 3 h into the L period. 11-day-old second leaves of *S. spontaneum* (SES-208) were cut into 15 1-cm segments, 15-day-old second leaves of *S. officinarum* (LA-purple) were cut into 15 1-cm segments. Samples were pooled from an average of four plants per biological replicate, and three biological replicates in total were prepared. The previously described approach by Li et al. was followed [50].

For the diurnal cycle experiment: Leaves from *Saccharum* were collected from a field at Fujian Agriculture and Forestry University (Fuzhou, Fujian, 119°16′48″E, 26°4′48″N) for RNA extraction. The mature leaves of *S. spontaneum* (SES-208) was collected from one plant as one replicate, and three biological replicates were collected every hour between 6 a.m. on March, 2 2017 and 4 a.m. on March 3, 2017, 12 time points (6 a.m., 8 a.m., 10 a.m., noon, 2 p.m., 4 p.m., 6 p.m., 8 p.m., 10 p.m., midnight, 2 a.m., 4 a.m.) were chosen for RNA-seq library construction. At the same time, a mature leaf was collected every hour between 6 a.m. on the 2nd March 2017 and 6 a.m. on the 3rd March 2017, seven time points (6 a.m., 10 a.m., 2 p.m., 6 p.m., 10 p.m., 2 a.m., 6 a.m.) were chosen for RNA-seq library construction. The collection of *S. officinarum* (LA-purple) leaves was performed in the same way. The sunset time on 2 March was 6 p.m. The previously described approach by Ming et al. was followed [43].

For the parenchyma and sclerenchyma cells experiment:We generated RNA from parenchyma cells and sclerenchyma cells isolated by hand sectioning from internode 13 of 11-month-old *S. officinarum* (LA-purple), which were grown in a field at Fujian Agriculture and Forestry University*.*

### BAC sequencing

A total of 38,400 BAC clones were constructed from *S. spontaneum* AP85–441 (2n = 4x = 32), a haploid clone of *S. spontaneum* following the method described by Ming et al. [34, 35, 84]*.*

### Homolog searches

The completely sequenced genomes and predicted protein sequences of plants were downloaded from Phytozome version 10.0 [39] and the National Center for Biotechnology Information (NCBI) databases [85]. To identify proteins containing *MtN3_slv* domains in the proteome datasets, the *MtN3_slv* domain PF03083 model profile from the Pfam database [86] was used to perform local searches in the four proteome datasets using the HMMER program [87].

### Phylogenetic analysis

The sequence annotation, functional prediction of *SsSWEETs* following the method described in previous studies [34, 35]. Based on the alignment of protein sequences, the phylogenetic tree of the *SWEET* gene family was constructed using neighbor-joining (NJ) and maximum likelihood (ML) methods. The construction of NJ tree was carried out using MEGA (version 5.0) [88] with the ‘pairwise deletion’ option and the ‘Poisson correction’ model [88]. Reliability of internal branches of the tree was valued by the bootstrapping of 1000 replicates. PhyML (version 3.0) was applied to construct a ML tree, with 100 nonparametric bootstrap replicates, γ-distribution, and WAG amino acid substitution model [89].

### Calculation of Ka/Ks

Ka/Ks ratios were calculated in KaKs_Calculator v2.0 using the maximum-likelihood MA method [90]. Meanwhile, Fisher’s exact test for small samples was applied to justify the validity of Ka and Ks calculated by this method [91]. The divergence time (T) was calculated by T = Ks/ (2 × 6.1 × 10^− 9^) × 10^− 6^ Mya [92].

### Analysis of the expression profiling of *SWEETs* in *Saccharum* based on RNA-seq

The cDNA libraries preparation was performed according to the manufacturer’s protocol (TruSeq® RNA, Illumina). The RNA-seq libraries were pooled and sequenced with 100 nt paired-end on an Illumina HiSeq2500 platform at the Center for Genomics and Biotechnology, Fujian Agriculture and Forestry University. Raw data were aligned to reference gene models (sorghum gene models) using TRINITY [93]. RNA-seq quantitative analysis was completed through Trinity Transcript Quantification and the RPKM value of the gene was calculated by RSEM method.

### Experimental validation of *SWEET* gene expression levels by RT-qPCR

The expression levels of two *SWEET* genes (*SsSWEET2* and *SsSWEET4b*) in five tissues (Stem 3, 9, 15, leaves and leaf roll in LA-Purple, and Stem 3, 6, 9, leaves and leaf roll in SES-208) from two 12-months old *Saccharum* species were validated by RT-qPCR (Additional file 7). The real time PCR reaction program and method to calculate the relative expression levels was performed as previously described [34, 35].

### Experimental validation of *SWEET16b* gene by PCR

Extraction of sugarcane genomic DNA was performed by the cetyltrimethyl ammonium bromide (CTAB) method. Gene-specific primer pairs (Additional file 8) were designed using the Primer 5 software. PCRs were performed using PrimeSTAR® Max DNA Polymerase (TakaRa, R045A). PCR reaction program: 95 °C for 3 min; 95 °C for 30s, 57 °C for 30s, 72 °C for 2 min, 34 cycles; 72 °C 5 min; 4 °C save.

## Additional files

Additional file 1: The *SWEET* gene alleles in *S. spontaneum*. (XLSX 12 kb)

Additional file 2: Percentage similarity between SWEET proteins in sugarcane was calculated using NCBI BLASTP software. (DOCX 23 kb)

Additional file 3: A schematic diagram for the relationship of the four clades of the phylogenetic tree constructed by the ML method. (PDF 42 kb)

Additional file 4: An unrooted tree using SWEET amino acid sequences from sugarcane, sorghum, maize and rice *SWEET* genes and the MtN3_slv domain architecture of those proteins. (PDF 2012 kb)

Additional file 5: **A** RT-qPCR verification of *SWEET2b* and *SWEET4b* in partial tissues of two *Saccharum* species. Note: IN, internode; LR, leaf roll. Internodes 3, 9, 15 and internodes 3, 6, 9 were from *S. officinarum* LA-Purple and *S. spontaneum* SES-208, respectively. **B** Correlation coefficient between RNA-seq (X axis) and RT-qPCR (Y axis) of two *SsSWEET* genes (*SsSWEET2b* and *SsSWEET4b*). (PDF 47 kb)

Additional file 6: PCR verification of *SWEET4e* and *SWEET16b* in two *Saccharum* species. (PDF 53 kb)

Additional file 7: The primers for RT-qPCR verification of *SWEET2b* and *SWEET4b* in two *Saccharum* species. (DOCX 17 kb)

Additional file 8: The primers for PCR verification of *SWEET4e* and *SWEET16b* in two *Saccharum* species. (DOCX 17 kb)

## Abbreviations

AMF: Arbuscular mycorrhizal fungi
BAC: Bacterial Artificial Chromosome
BETL: Basal endosperm transfer layer
CDS: Coding domain sequence
CTAB: Cetyltrimethyl ammonium bromide
CWINVs: Cell-wall invertases
DW: Dry weight
eEF-1a: Eukaryotic elongation factor 1a
ESTs: Expressed sequence tags
GAPDH: Glyceraldehyde-3-phosphate dehydrogenase
Ka/Ks: Nonsynonymous to synonymous substitution ratio
LCA: Last common ancestor
MSTs: MonoSaccharide Transporters
ORFs: Open reading frames
PCR: Polymerase chain reaction
RNA-seq: RNA Sequencing
RPKM: Reads Per Kilobase per Million mapped reads
RT-qPCR: Reverse transcription quantitative PCR
7-TM: seven predicted transmembrane
SUTs: Sucrose Transporters/Sucrose Carriers
SWEET: Sugars Will Eventually be Exported Transporters
THB: Triple-helix bundles
TM: Transmembrane domain
VINVs: Vacuolar invertases
WGD: Whole genome duplication
