# Supplementary material for: New insights into the evolution and functional divergence of the SWEET family in Saccharum based on comparative genomics
Source: BMC Plant Biol. 2018 Nov 7;18:270. doi: 10.1186/s12870-018-1495-y (PMC6222987; doi:10.1186/s12870-018-1495-y)
Supplement: Supplementary file 2 — Percentage similarity between SWEET proteins in sugarcane was calculated using NCBI BLASTP software. (DOCX 23 kb) [file 12870_2018_1495_MOESM2_ESM.docx]

**Additional File 2**. Percentage of similarity between SWEET proteins in sugarcane was calculated using NCBI BLASTP.

|  | 1a | 1b | 2a | 2b | 3a | 3b | 4a | 4b | 4c | 4d | 5 | 6 | 11a | 11b | 12 | 13a | 13b | 13c | 14 | 15 | 16a |
| --- | --- | --- | --- | --- | --- | --- | --- | --- | --- | --- | --- | --- | --- | --- | --- | --- | --- | --- | --- | --- | --- |
| SsSWEET1b | 69% |  |  |  |  |  |  |  |  |  |  |  |  |  |  |  |  |  |  |  |  |
| SsSWEET2a | 47% | 51% |  |  |  |  |  |  |  |  |  |  |  |  |  |  |  |  |  |  |  |
| SsSWEET2b | 39% | 41% | 53% |  |  |  |  |  |  |  |  |  |  |  |  |  |  |  |  |  |  |
| SsSWEET3a | 36% | 38% | 36% | 31% |  |  |  |  |  |  |  |  |  |  |  |  |  |  |  |  |  |
| SsSWEET3b | 38% | 33% | 38% | 33% | 30% |  |  |  |  |  |  |  |  |  |  |  |  |  |  |  |  |
| SsSWEET4a | 43% | 44% | 43% | 35% | 48% | 33% |  |  |  |  |  |  |  |  |  |  |  |  |  |  |  |
| SsSWEET4b | 45% | 45% | 42% | 37% | 49% | 33% | 93% |  |  |  |  |  |  |  |  |  |  |  |  |  |  |
| SsSWEET4c | 43% | 42% | 43% | 38% | 48% | 32% | 83% | 84% |  |  |  |  |  |  |  |  |  |  |  |  |  |
| SsSWEET4d | 37% | 39% | 34% | 36% | 28% | 50% | 32% | 31% | 31% |  |  |  |  |  |  |  |  |  |  |  |  |
| SsSWEET5 | 47% | 46% | 43% | 37% | 39% | 34% | 54% | 58% | 49% | 36% |  |  |  |  |  |  |  |  |  |  |  |
| SsSWEET6 | 40% | 39% | 34% | 28% | 35% | 29% | 48% | 51% | 47% | 30% | 57% |  |  |  |  |  |  |  |  |  |  |
| SsSWEET11a | 38% | 36% | 33% | 31% | 31% | 28% | 29% | 29% | 27% | 31% | 36% | 30% |  |  |  |  |  |  |  |  |  |
| SsSWEET11b | 37% | 34% | 35% | 35% | 32% | 34% | 34% | 36% | 33% | 34% | 35% | 30% | 69% |  |  |  |  |  |  |  |  |
| SsSWEET12 | 36% | 36% | 33% | 31% | 35% | 30% | 34% | 34% | 30% | 34% | 34% | 30% | 51% | 53% |  |  |  |  |  |  |  |
| SsSWEET13a | 35% | 36% | 34% | 31% | 28% | 34% | 35% | 36% | 33% | 34% | 35% | 33% | 55% | 53% | 54% |  |  |  |  |  |  |
| SsSWEET13b | 38% | 36% | 34% | 31% | 28% | 33% | 35% | 37% | 33% | 33% | 35% | 33% | 55% | 53% | 55% | 95% |  |  |  |  |  |
| SsSWEET13c | 37% | 37% | 33% | 31% | 27% | 32% | 36% | 36% | 32% | 32% | 36% | 32% | 54% | 52% | 54% | 93% | 94% |  |  |  |  |
| SsSWEET14 | 37% | 37% | 37% | 31% | 28% | 28% | 35% | 36% | 31% | 31% | 32% | 29% | 51% | 51% | 61% | 68% | 68% | 73% |  |  |  |
| SsSWEET15 | 41% | 40% | 37% | 35% | 32% | 31% | 35% | 36% | 33% | 33% | 37% | 32% | 54% | 55% | 53% | 59% | 63% | 58% | 62% |  |  |
| SsSWEET16a | 47% | 49% | 40% | 34% | 36% | 32% | 42% | 42% | 37% | 35% | 46% | 36% | 41% | 42% | 43% | 44% | 43% | 43% | 43% | 48% |  |
| SsSWEET16b | 42% | 45% | 41% | 36% | 34% | 33% | 41% | 41% | 37% | 33% | 43% | 33% | 35% | 39% | 34% | 40% | 40% | 37% | 37% | 41% | 48% |
