# Supplementary figures and images for: New insights into the evolution and functional divergence of the SWEET family in Saccharum based on comparative genomics
Source: BMC Plant Biol. 2018 Nov 7;18:270. doi: 10.1186/s12870-018-1495-y (PMC6222987; doi:10.1186/s12870-018-1495-y)

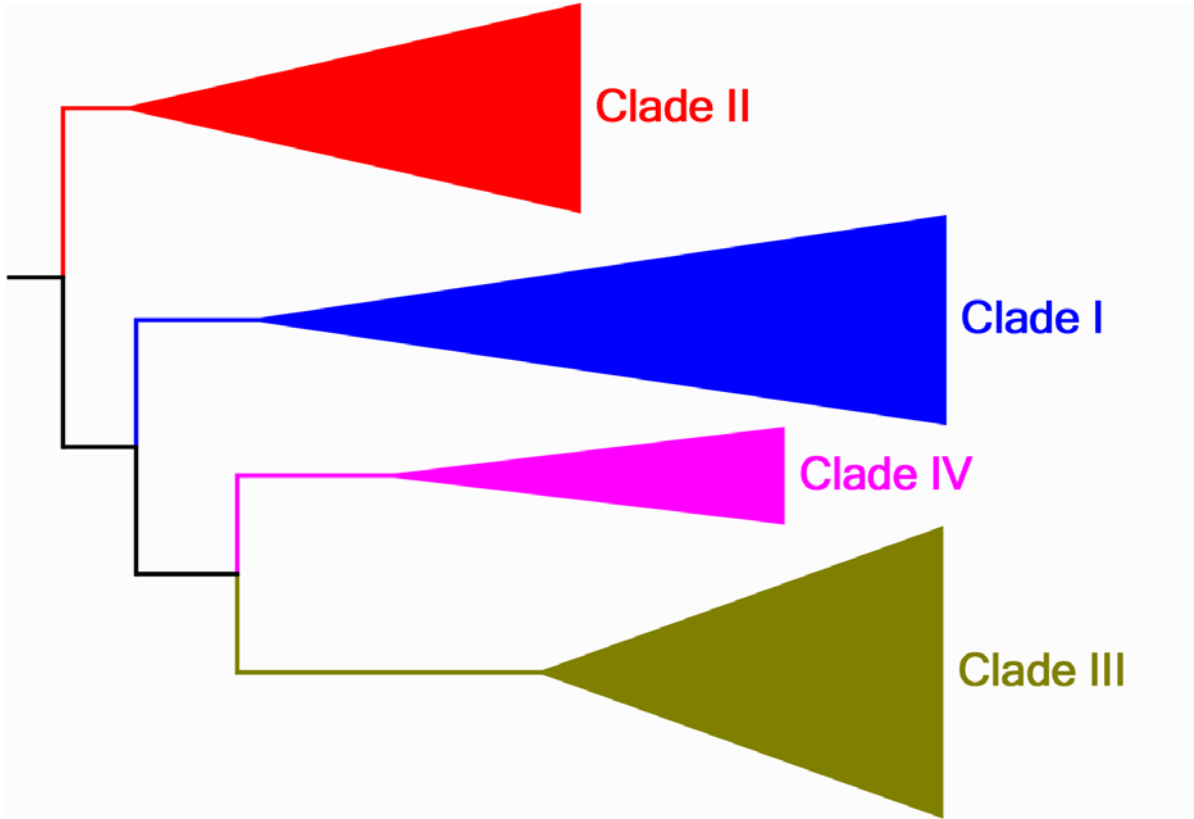

Supplement: Supplementary file 3 — A schematic diagram for the relationship of the four clades of the phylogenetic tree constructed by the ML method. (PDF 42 kb) [file 12870_2018_1495_MOESM3_ESM.pdf]

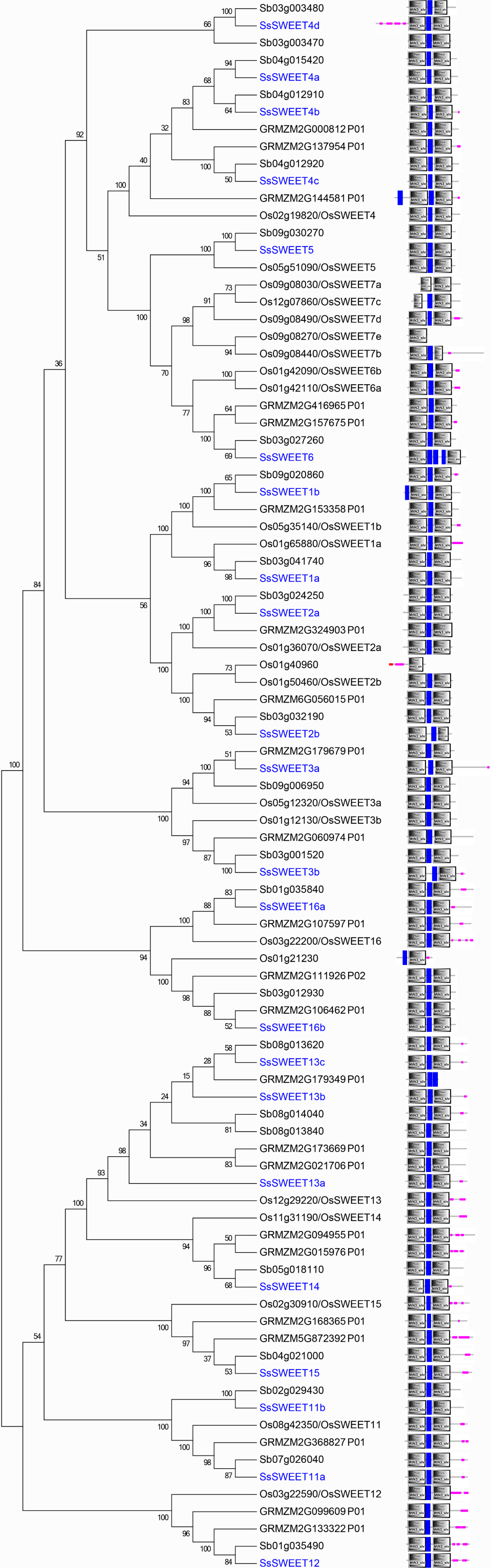

Supplement: Supplementary file 4 — An unrooted tree using SWEET amino acid sequences from sugarcane, sorghum, maize and rice SWEET genes and the MtN3_slv domain architecture of those proteins. (PDF 2012 kb) [file 12870_2018_1495_MOESM4_ESM.pdf]

A

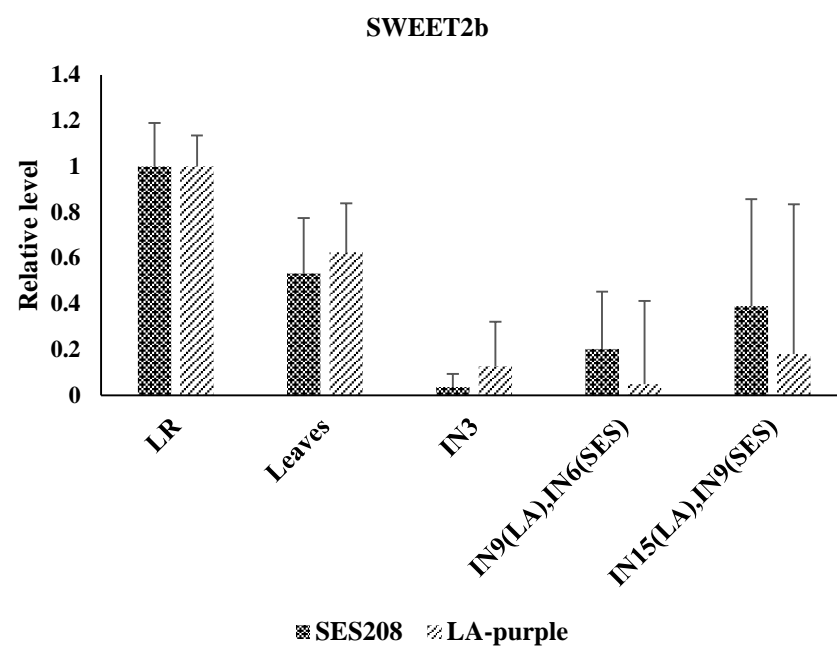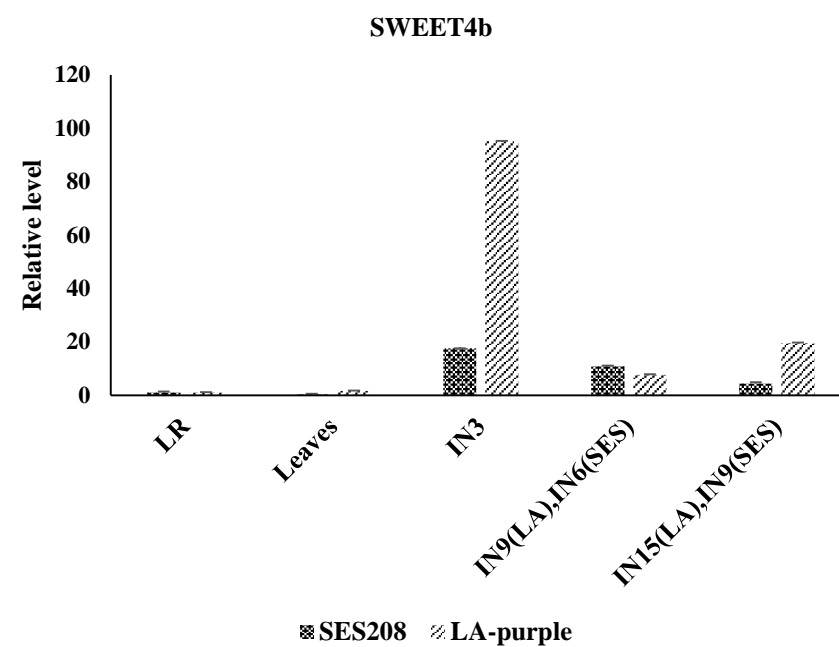

B

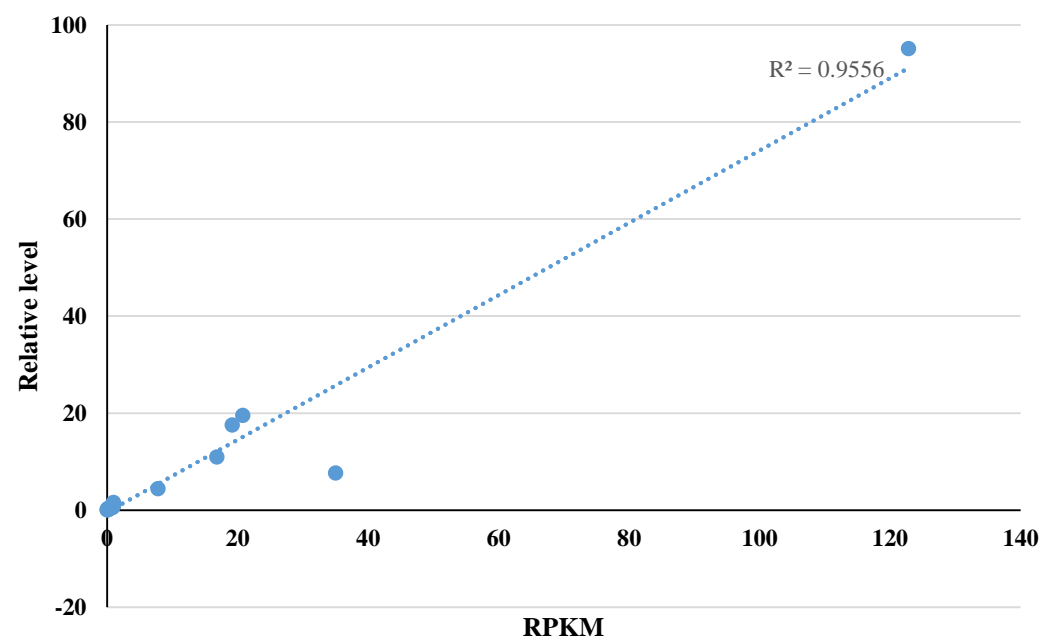

Supplement: Supplementary file 5 — A RT-qPCR verification of SWEET2b and SWEET4b in partial tissues of two Saccharum species. Note: IN, internode; LR, leaf roll. Internodes 3, 9, 15 and internodes 3, 6, 9 were from S. officinarum LA-Purple and S. spontaneum SES-208, respectively. B Correlation coefficient between RNA-seq (X axis) and RT-qPCR (Y axis) of two SsSWEET genes (SsSWEET2b and SsSWEET4b). (PDF 47 kb) [file 12870_2018_1495_MOESM5_ESM.pdf]

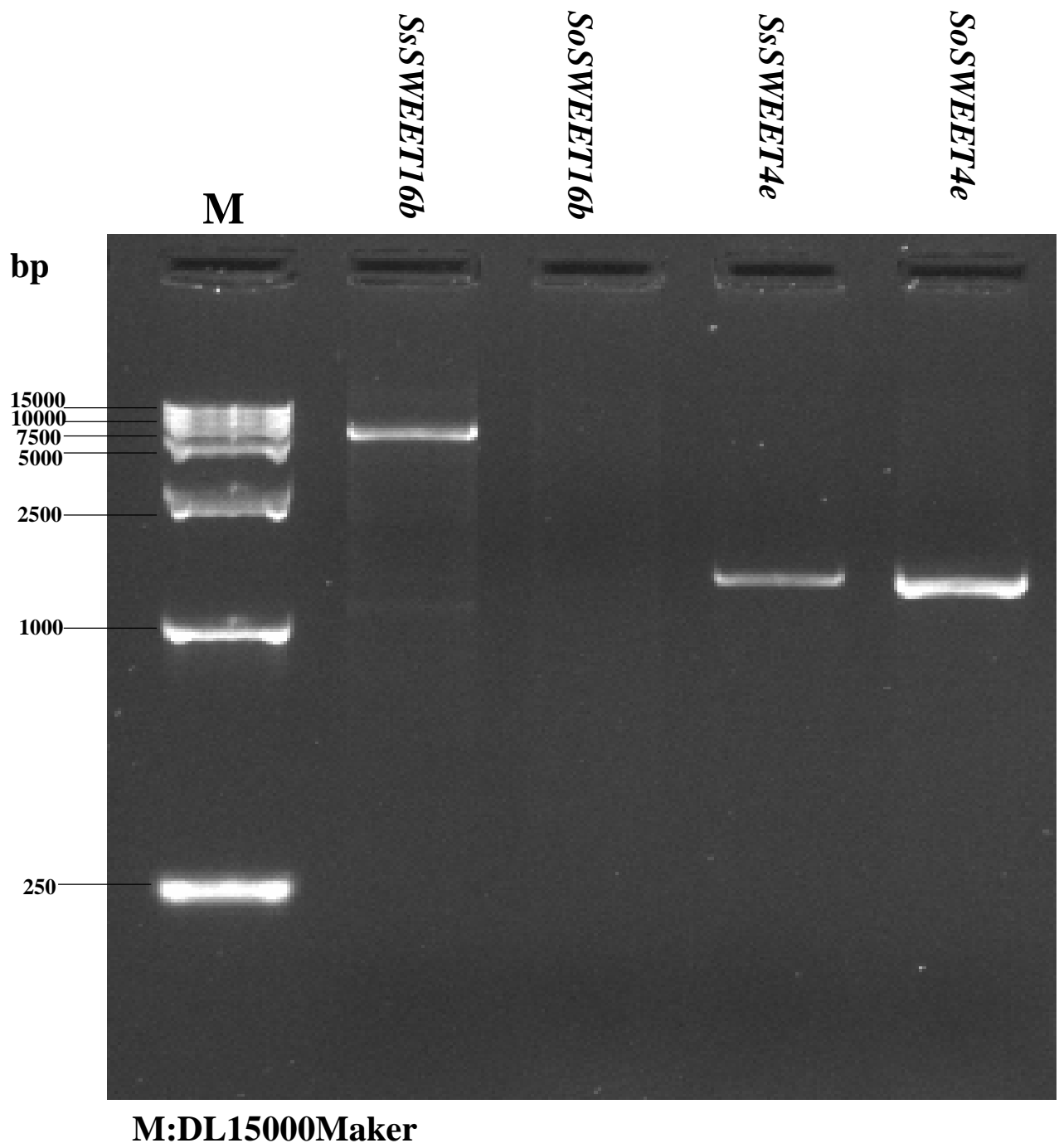

Supplement: Supplementary file 6 — PCR verification of SWEET4e and SWEET16b in two Saccharum species. (PDF 53 kb) [file 12870_2018_1495_MOESM6_ESM.pdf]
