# Supplementary material for: New insights into the evolution and functional divergence of the SWEET family in Saccharum based on comparative genomics
Source: BMC Plant Biol. 2018 Nov 7;18:270. doi: 10.1186/s12870-018-1495-y (PMC6222987; doi:10.1186/s12870-018-1495-y)
Supplement: Supplementary file 7 — The primers for RT-qPCR verification of SWEET2b and SWEET4b in two Saccharum species. (DOCX 17 kb) [file 12870_2018_1495_MOESM7_ESM.docx]

**Additional File 7.** The primers for RT-qPCR verification of *SWEET2b* and *SWEET4b* in two *Saccharum* species

| Gene name | Forward primer(5’-3’) | Reverse primer(5’-3’) |
| --- | --- | --- |
| *SWEET2b* | GCCTACATCTCGCTCTTCATC | GAAGACCACTAGCACCAGAAG |
| *SWEET4b* | CATCCACTTCGACCTCTACATC | CGATGATCTTCTGGGTGTTCTT |
