# Supplementary material for: New insights into the evolution and functional divergence of the SWEET family in Saccharum based on comparative genomics
Source: BMC Plant Biol. 2018 Nov 7;18:270. doi: 10.1186/s12870-018-1495-y (PMC6222987; doi:10.1186/s12870-018-1495-y)
Supplement: Supplementary file 8 — The primers for PCR verification of SWEET4e and SWEET16b in two Saccharum species. (DOCX 17 kb) [file 12870_2018_1495_MOESM8_ESM.docx]

**Additional File 8**. The primers for PCR verification of *SWEET4e* and *SWEET16b* in two *Saccharum* species

| Gene name | Forward primer(5’-3’) | Reverse primer(5’-3’) |
| --- | --- | --- |
| *SWEET4e* | ATGGGAGCCCAGGAGATCTCC | CAAGACGAAGAGGTCGGAGCC |
| *SWEET16b* | CCTAGCTATAATTGCACTGC | GCTAGATGCTTCTCCCTGAT |
